# Pro-Inflammatory Cytokines Promote the Transcription of Circular RNAs in Human Pancreatic β Cells

**DOI:** 10.3390/ncrna8050069

**Published:** 2022-10-12

**Authors:** Simranjeet Kaur, Caroline Frørup, Aashiq H. Mirza, Tina Fløyel, Reza Yarani, Maikel L. Colli, Jesper Johannesen, Joachim Størling, Decio L. Eizirik, Flemming Pociot

**Affiliations:** 1 Translational Type 1 Diabetes Research, Department of Clinical Research, Steno Diabetes Center Copenhagen, 2730 Herlev, Denmark; 2 Department of Pharmacology, Weill Cornell Medical, New York, NY 10065, USA; 3 ULB Center for Diabetes Research, Medical Faculty, Université Libre de Bruxelles, 1070 Brussels, Belgium; 4 Department of Paediatrics and Adolescent Medicine, Herlev and Gentofte Hospital, Copenhagen University Hospital, 2730 Herlev, Denmark; 5 Faculty of Health and Medical Sciences, University of Copenhagen, 2200 Copenhagen, Denmark; 6 Department of Biomedical Sciences, Faculty of Health and Medical Sciences, University of Copenhagen, 2200 Copenhagen, Denmark

**Keywords:** non-coding RNA, type 1 diabetes, circRNA, miRNA, human islets, microarray, inflammation, *CRIM1*, *WARS*, *EPSTI1*

## Abstract

Circular RNAs (circRNAs) have recently been implicated in impaired β-cell function in diabetes. Using microarray-based profiling of circRNAs in human EndoC-βH1 cells treated with pro-inflammatory cytokines, this study aimed to investigate the expression and possible regulatory roles of circRNAs in human β cells. We identified ~5000 β-cell-expressed circRNAs, of which 84 were differentially expressed (DE) after cytokine exposure. Pathway analysis of the host genes of the DE circRNAs revealed the enrichment of cytokine signaling pathways, indicative of circRNA transcription from inflammatory genes in response to cytokines. Multiple binding sites for β-cell-enriched microRNAs and RNA-binding proteins were observed for the highly upregulated circRNAs, supporting their function as ‘sponges’ or ‘decoys’. We also present evidence for circRNA sequence conservation in multiple species, the presence of cytokine-induced regulatory elements, and putative protein-coding potential for the DE circRNAs. This study highlights the complex regulatory potential of circRNAs, which may play a crucial role during immune-mediated β-cell destruction in type 1 diabetes.

## 1. Introduction

Pro-inflammatory cytokines, such as interleukin (IL)-1β and interferon (IFN)-γ secreted from activated immune cells, play a crucial role in islet inflammation and β-cell dysfunction in type 1 diabetes (T1D) [1,2]. Cytokines exert their detrimental effects on the β cells via modulating the gene expression, induction of endoplasmic reticulum stress, and activation of the apoptosis pathway, ultimately leading to impaired insulin secretion and β-cell apoptosis [3].

Non-coding RNAs (ncRNA) have emerged as essential players in the regulation of gene expression, and their dysregulation contributes to various pathophysiological conditions [4,5]. In the last decade, ncRNAs, including microRNAs (miRNA) and long non-coding RNAs (lncRNA), have been implicated in pancreatic β-cell dysfunction and apoptosis in T1D [6,7,8,9]. Circular RNAs (circRNAs) are a novel class of ncRNAs with a covalent closed continuous loop, i.e., the 5′ and 3′ termini are covalently linked by an unconventional type of alternative splicing known as back-splicing of exons from a single pre-mRNA [10,11,12]. In recent years, several studies have shown that circRNAs regulate a range of cellular functions, affecting biological processes in the body [4,13]. Their proposed molecular mechanisms of action include modulating gene regulation by interacting with RNA-binding proteins or serving as decoys for miRNAs [14,15,16].

Several circRNAs have been reported to be associated with T1D [17,18,19,20,21,22,23]. Li et al. identified 68 differentially expressed plasma circRNAs in individuals with new-onset T1D compared to healthy controls [19]. Others have reported several circRNAs to be differentially expressed in peripheral blood mononuclear cells (PBMCs) from individuals with T1D compared to healthy controls [20,21,22]. A recent study by Wang et al. reported around 2000 differentially expressed circRNAs in the mouse β-cell line MIN6 after pro-inflammatory cytokine exposure [23]. Many circRNAs have also been identified in human pancreatic islets and β cells [17,24,25]. Haque et al. and Stoll et al. both identified thousands of circRNAs expressed in human islets by CircleSeq and microarray analysis, respectively [17,25]. Stoll et al. showed that almost 500 of the identified circRNAs were conserved in mouse islets [17]. Using publicly available datasets, we previously explored the cell-type-specific expression of circRNAs in human α, β, and exocrine cells and demonstrated that circRNAs are abundantly and selectively expressed in β cells [24]. 

The molecular mechanisms of circRNAs in T1D are not yet well-understood. However, there is increasing evidence for their involvement in the regulation of β-cell function [17,18,26]. Reduced expression of two circRNAs, circHIPK3 and ciRS-7/CDR1, was observed in the islets of diabetic db/db mice [17]. Forced downregulation of these transcripts in the islets of wild-type animals resulted in reduced insulin secretion, β-cell proliferation, and survival. circHIPK3 acted by sequestering a group of miRNAs, such as miR-124-3p and miR-338-3p, and by regulating the expression of key β-cell genes, including *SLC2A2, AKT1,* and *MTPN* [17]. ciRS-7/CDR1 was previously proposed to function by sponging miR-7 and regulating insulin secretion and β-cell proliferation [26]. These findings suggest that specific circRNAs may play roles in β-cell function and survival and infer their possible involvement in T1D. 

In the present study, we explored the regulation of circRNAs by pro-inflammatory cytokines in human EndoC-βH1 cells and isolated human islets to shed light on their potential roles in β-cell dysfunction in T1D pathogenesis.

## 2. Materials and Methods

### 2.1. Cell and Islet Culture 

EndoC-βH1 cells [27], kindly provided by Dr. Raphael Scharfmann, were maintained in Dulbecco’s Modified Eagle Medium (DMEM) with 5.6 mmol/L glucose in 2% fatty-acid-free bovine serum albumin (BSA) fraction V, 50 µmol/L 2-mercaptoethanol (all from Gibco, Thermo-Fisher Scientific, Waltham, MA, USA), 10 mmol/L nicotinamide, 5.5 µg/mL transferrin, 6.7 ng/mL selenite (all from Sigma-Aldrich, St. Louis, MO, USA), 100 U/mL penicillin, and 100 µg/mL streptomycin on matrigel–fibronectin-coated plates, as previously described [28].

Isolated human pancreatic islets from four islet donors were purchased from Prodo Laboratories Inc. via Tebu-Bio. See Supplementary Materials for donor information (Supplementary Table S1). Islets were maintained in an F-10 Nutrient Mix medium with GlutaMAX supplemented with 10% fetal bovine serum (FBS), 100 U/mL penicillin, and 100 µg/mL streptomycin. 

Both human islets and EndoC-βH1 cells were exposed to the following cytokine concentrations based on previous dose–response experiments performed by our group [3]: 50 U/mL recombinant human IL-1β (R&D Systems, Minneapolis, MN, USA) and 1000 U/mL recombinant human IFN-γ (PeproTech, Cranbury, NJ, USA) for 24 h or 48 h and compared to untreated islets and cells.

### 2.2. circRNA Microarray Labeling and Hybridization

Total RNA from EndoC-βH1 cells was extracted and quantified using a NanoDrop ND-1000 as described previously [29]. The sample preparation and microarray hybridization were performed based on the Arraystar’s standard protocols. Briefly, total RNAs were digested with RNase R (Epicentre, Inc., Madison, WI, USA) to remove linear RNAs and enrich circular RNAs. Then, the enriched circular RNAs were amplified and transcribed into fluorescent cRNA utilizing a random priming method (Arraystar Super RNA Labeling Kit; Arraystar, Rockville, MD, USA). The labeled cRNAs were purified by an RNeasy Mini Kit (Qiagen, Hilden, Germany) and hybridized onto the Arraystar Human circRNA Array V2 (8 × 15 K, Arraystar). The concentration and specific activity of the labeled cRNAs (pmol Cy3/µg cRNA) was measured by a NanoDrop ND-1000. Of each labeled cRNA, 1 µg was fragmented by adding 5 µL of 10 × Blocking Agent and 1 µL of 25 × Fragmentation Buffer, then heated at 60 °C for 30 min. Finally, 25 µL of 2 × Hybridization Buffer was added to dilute the labeled cRNA. Next, 50 µL of hybridization solution was dispensed into the gasket slide and assembled into the circRNA expression microarray slide. The slides were incubated for 17 h at 65 °C in an Agilent Hybridization Oven (Agilent Technologies, Santa Clara, CA, USA). The hybridized arrays were washed, fixed, and scanned using an Agilent G2505C Scanner. Agilent Feature Extraction software (version 11.0.1.1) was used to analyze the acquired array images.

### 2.3. Differential Expression Analysis

Quantile normalization and subsequent data processing were performed using the R software limma package. After the quantile normalization of the raw data, low-intensity filtering was performed, and circRNAs with flags “P” or “M” (“All Targets Value”) in at least 3 out of 12 samples were retained for further analyses. Differentially expressed (DE) circRNAs with statistical significance between the two groups were identified using an absolute log fold change (log(FC)) cutoff of 1 and an adjusted *p*-value (adj. *p*-value) < 0.05. The DE circRNAs were visualized as volcano plots. Hierarchical clustering was performed to show the distinguishable circRNA expression pattern among the samples.

### 2.4. Real-Time qPCR Validation of circRNAs, Host Genes, miRNA Targets, and miRNAs

Three upregulated circRNAs (circWARS, circEPSTI1, and circCRIM1) with high expression levels were selected for further validation in human EndoC-βH1 cells and human pancreatic islets. The expression levels of circRNAs, host genes, miRNA targets, and miRNAs were determined by real-time qPCR and quantified using the 2^−ΔΔCt method [30]. RNA was extracted using an RNeasy Mini Kit (Qiagen) or a Direct-zol RNA Miniprep Kit (Zymo Research, Irvine, CA, USA). circRNAs were enriched with 20 U/1000 ng RNA of RNase R treatment (Lucigen, Middleton, WI, USA) and the RNA clean-up protocol from the RNeasy Mini Kit (Qiagen). The preparation of cDNA was carried out using the iScript cDNA Synthesis Kit (Bio-Rad, Hercules, CA, USA), the TaqMan Advanced miRNA cDNA Synthesis Kit (Applied Biosystems, Waltham, MA, USA), or the iScript Select Synthesis kit (Bio-Rad) with random hexamer primers. Real-time qPCR was performed on a CFX384 system (Bio-Rad). The expression levels of host genes and miRNA target genes were evaluated using TaqMan Assays and TaqMan Gene Expression Master Mix (Applied Biosystems). miRNAs were evaluated using TaqMan Advanced miRNA assays and Taqman Fast Advanced Master Mix (Applied Biosystems), and circRNAs were evaluated with PrimeTime Assays (Integrated DNA Technologies, Coralville, IA, USA) and PowerUp SYBR Green Master Mix (Applied Biosystems). CircRNAs were detected using divergent primers with back-splice-junction-spanning probes. The primer sequences for the selected circRNAs are listed in Supplementary Table S2. Host mRNA and circRNA expression were normalized using *GAPDH,* and miRNA expression was normalized using hsa-miR-375 as an internal control.

### 2.5. RNA Sequencing and Proteomics Data in EndoC-βH1 Cells 

The host gene and protein expression in EndoC-βH1 cells previously published by our group [29] was retrieved from the GEO database (GSE137136) and ProteomeXchange (PXD011902), respectively. The spliced sequences for the DE circRNAs were retrieved from the circBase database [31] to predict the potential ORFs and the peptide sequences. Only 72 circRNAs with circBase IDs were used in this analysis. The potential ORFs in each spliced sequence were identified, and their amino acid sequences were determined using the EMBOSS getORF app “http://emboss.sourceforge.net/apps/cvs/emboss/apps/getorf.html (accessed on 29 June 2020)” with the following parameters: minimum nucleotide size of ORF to report: 30; maximum nucleotide size of ORF to report: 1,000,000; output: translation of regions between start and stop codons; circular sequence: yes; find ORFs in reverse sequence: yes; number of flanking nucleotides to report: 100. 

### 2.6. Prediction of circRNA–miRNA and circRNA–RNA-Binding Protein Interactions

circRNA–miRNA interactions were predicted using StarBase database v3 [32]. We used published CLIPseq data for miRNAs retrieved from the StarBase database to identify miRNA binding sites for the DE circRNAs. All miRNA–target interactions were identified using the TargetScan [33] and MirTarBase [34] databases in the CyTargetLinker app in Cytoscape [35,36]. Human β-cell-enriched miRNAs were retrieved from [37]. The CircInteractome database [38], which uses 93 independently reported CLIP datasets from various RBPs obtained from different tissues and cell lines, was used for predicting RNA binding sites on circRNA junctions and junction-flanking sequences.

### 2.7. Pathway Analysis

Pathway analysis was performed using WikiPathways, KEGG, and Reactome annotations in the ClueGO v2.5.4 app in Cytoscape [36,39]. ClueGO calculates over-represented GO terms and pathways using the two-sided hypergeometric test and merges GO and pathway terms with a minimum of 50% overlapping genes into clusters. The *p*-values were adjusted using the Bonferroni step-down method, and a Kappa score threshold of 0.4 was used for making clusters. All unique genes (*n* = 12,815) from WikiPathways, KEGG, and Reactome were used as the reference set for the pathway analysis. This was performed because an ideal gene set with only the genes associated with the circRNAs was not available due to incomplete annotations.

### 2.8. circRNAs Enriched in IRES and IRE Sites

IRES-like short elements that are significantly enriched in circRNAs were retrieved from [40]. The exonic circRNA sequences were screened against the library of IRES elements to predict their translation potential. The DE circRNAs were intersected with cytokine-induced regulatory element (IRE) datasets downloaded from the Islet Regulome Browser “http://isletregulome.com (accessed on 4 May 2020)” and [29]. 

### 2.9. Evolutionarily Conserved circRNAs 

Evolutionary conservation can be used as a guide to indicate regions of non-coding or coding DNA that are likely to have a biological function and thus may be more likely to harbor SNP variants with functional consequences. We used a multi-species conserved sequences (MCS) analysis using the CircAtlas database [41] to prioritize circRNAs for follow-up and candidate gene association studies.

## 3. Results

### 3.1. circRNAs Are Differentially Expressed in the EndoC-βH1 β-Cell Line

**Figure 1:**
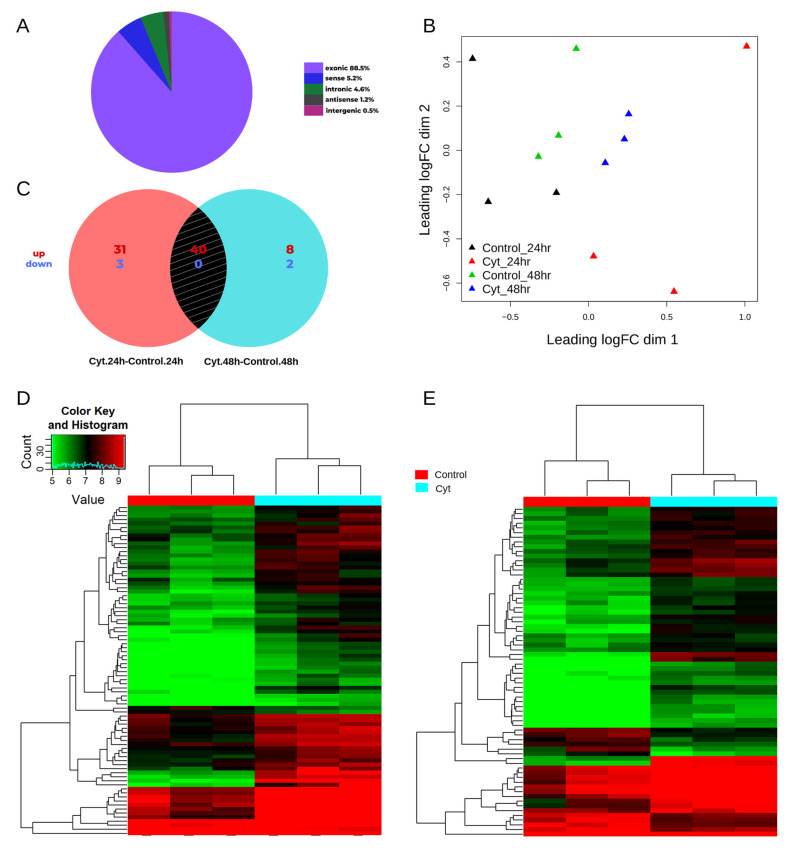
CircRNAs modulated by pro-inflammatory cytokines in human β cells. (**A**) Distribution of circRNAs expressed in human EndoC-βH1 cells based on their biotypes; (**B**) MDS plot showing the effect of cytokine-treatment at different time-points in EndoC-βH1 samples; (**C**) Venn diagram showing the overlap between the number of DE circRNAs after cytokine exposure at 24 h and 48 h; (**D**,**E**) Hierarchical clustering of DE circRNAs based on their normalized expression values at 24 h (**D**) and 48 h (**E**) after cytokine exposure.

Human EndoC-βH1 cells were left untreated or exposed to the pro-inflammatory cytokines IL-1β and IFN-γ for 24 h or 48 h (*n* = 3 in each condition). An Arraystar Human CircRNA microarray V2 was used to profile the enriched circRNAs after an RNase R treatment of the total RNA. The array consisted of 13,617 probes targeting circRNA-specific junctions. After filtering for low-intensity probes, 10,206 probes remained for further analysis. These 10,206 circRNA probes expressed in EndoC-βH1 cells mapped to 5039 genes. As shown in Figure 1A, the majority of the expressed circRNAs were exonic (88%), while the rest mapped within introns (5%), sense-overlapping (5%) and antisense transcripts (1%), and intergenic regions (1%) of the genome. Multi-dimensional scaling showed a clear separation of samples based on the treatment, as seen in Figure 1B. 

**Figure 2:**
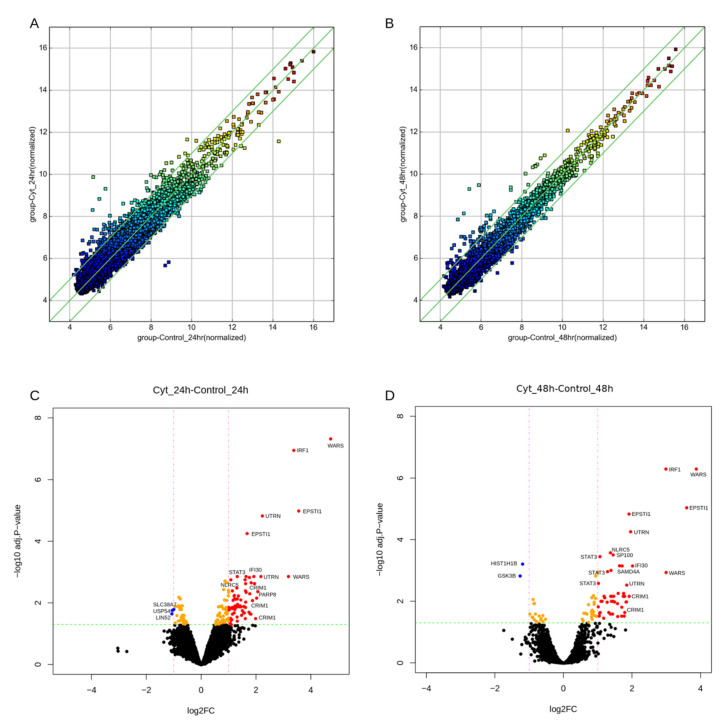
Comparison of circRNA expression profiles between cytokine-treated and non-treated samples at 24 h and 48 h in EndoC-βH1 cells. Scatter plots showing the differences in the expression of circRNAs in cytokine-treated samples compared to control samples at 24 h (**A**) and 48 h (**B**). The middle green line represents no difference between the two groups, while the flanking green lines indicate a change of 2-fold. The circRNAs beyond these lines represent > 2-fold changes between the two groups. Volcano plots of DE circRNAs in human EndoC-βH1 cells after cytokine exposure at 24 h (**C**) and 48 h (**D**). The horizontal and vertical lines represent the adj. *p*-value and fold-change cutoffs, respectively. The red and blue dots represent the upregulated and downregulated circRNA isoforms, respectively.

The differential expression analysis identified 74 and 50 circRNAs that were modulated after cytokine exposure for 24 h and 48 h, respectively (Figure 1C and Figure 2). There was an extensive overlap in the cytokine-modulated circRNAs between the two time-points (Figure 1C), resulting in a total of 84 DE circRNAs. These 84 DE circRNAs mapped to 50 host genes (46 protein-coding and 4 lincRNA genes) based on the GRCh37 genome annotation. These circRNAs were mostly exonic (86%), but a few intronic (7%) and sense-overlapping (7%) biotypes were also observed. 

The majority of the DE circRNAs were upregulated by cytokines, including 71 out of 74 and 48 out of 50 at 24 h and 48 h, respectively (Figure 1C). Among these, 40 circRNA isoforms originating from 18 host genes were common and upregulated at both 24 h and 48 h (Table 1). The hierarchical clustering of the normalized expression of the DE circRNAs showed a clear separation between the two groups at 24 h and 48 h (Figure 1D,E). Only five circRNAs were downregulated by cytokines (Supplementary Table S3). Two circRNA isoforms from the WARS locus were the most highly upregulated, followed by circRNAs at the *EPSTI1*, *IRF1, UTRN,* and *CRIM1* loci (Figure 2; Table 1). Interestingly, *CRIM1* gives rise to more than 100 circular isoforms, as reported in the circBase database [31], whereas only 4 protein-coding isoforms have been confirmed by the Ensembl project “https://www.ensembl.org/Homo_sapiens/Gene/Summary?db=core;g=ENSG00000150938 (accessed on 4 June 2020)”. Of the 19 circular isoforms tested on the array for *CRIM1*, 17 were upregulated by cytokine exposure (Table 1). 

### 3.2. Validation of circWARS, circEPSTI1, and circCRIM1 in EndoC-βH1 Cells and Human Pancreatic Islets

**Figure 3:**
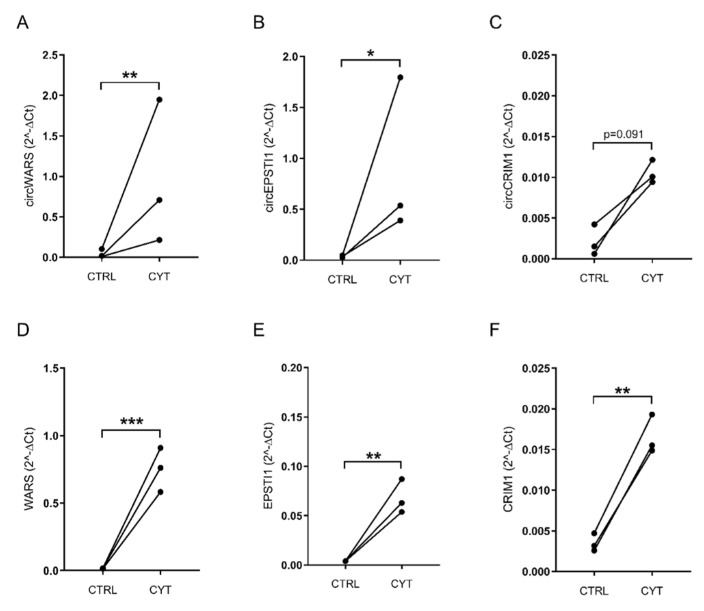
Validation of host genes and circRNAs of *WARS*, *EPSTI1,* and *CRIM1*. The expression of (**A**) circWARS, (**B**) circEPSTI1, and (**C**) circCRIM1 and the mRNA expression of (**D**) *WARS*, (**E**) *EPSTI1,* and (**F**) *CRIM1* in the EndoC-βH1 cells that were untreated (CTRL) or treated with IL-1β and IFN-γ (CYT) for 48 h (*n* = 3). The normalized expression values are presented as 2^−ΔCt. *GAPDH* was used as housekeeping gene. * *p* < 0.05, ** *p* < 0.01, *** *p* < 0.001.

Initially, we selected the three most upregulated circRNAs, hsa_circ_0033184 (circWARS), hsa_circ_0000479 (circEPSTI1), and hsa_circ_0001526 (circIRF1), for validation by real-time qPCR in EndoC-βH1 cells and isolated pancreatic islets from human donors treated or not treated with pro-inflammatory cytokines. However, due to a low basal expression level of hsa_circ_0001526 (circIRF1), this circRNA was excluded from the validation. hsa_circ_0002938 (circCRIM1) from the *CRIM1* locus (the most highly upregulated circRNA out of the 17 isoforms) was also selected for validation (Table 1). The upregulation of all three circRNAs, namely circWARS, circEPSTI1, and circCRIM1, was confirmed in EndoC-βH1 cells (Figure 3A–C; Supplementary Figure S1). circWARS and circEPSTI1 were also expressed in human islets (Supplementary Figure S2). circCRIM1 was lowly expressed and was only detected in two out of the four islet donors (data not shown). Furthermore, Sanger sequencing of the PCR products confirmed the back-spliced junctions for all three circRNAs in both the cytokine-treated and control samples in EndoC-βH1 cells (Supplementary Figure S3). We also investigated the expression of the host genes *WARS, EPSTI1,* and *CRIM1* and report that they were differentially expressed by cytokines in EndoC-βH1 cells in the same direction as the circRNAs (Figure 3D,E; Supplementary Figure S4). *WARS* and *EPSTI1* were also differentially expressed in human pancreatic islets (Supplementary Figure S4). 

### 3.3. Host Genes of the Cytokine-Regulated circRNAs Are Enriched in Interferon and Interleukin Signaling Pathways

**Figure 4:**
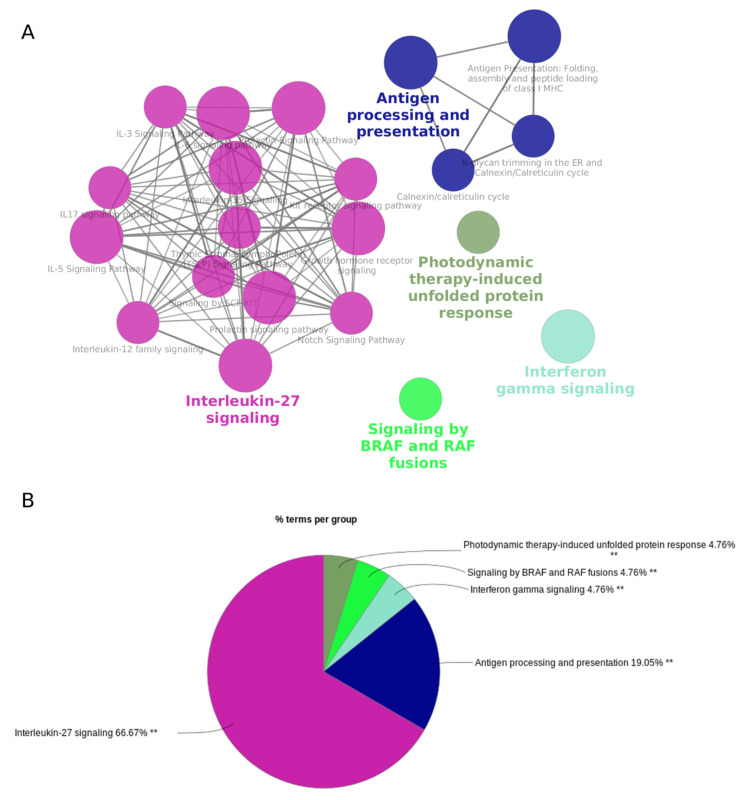
Network and pathway analysis of the DE circRNA host genes. (**A**) Network analysis based on pathway-based annotation of the 50 host genes of the DE circRNAs resulted in 21 significant pathways clustered into five groups. One selected term for each group is highlighted in bold. (**B**) Pie-chart representation of the above network highlights the five clusters (in different colors). The percentages shown for each cluster highlight their total contributions to the network based on the number of individual enriched pathway terms in each cluster. ** *p* < 0.01.

CircRNAs have been linked to the host genes and their function [42]. Based on this assumption, we performed a pathway analysis of the 50 host genes of the 84 DE circRNAs. The pathway analysis identified 21 enriched pathways that were grouped into five clusters. The most significant pathways for each of these five groups were: interleukin-27 signaling, interferon gamma signaling, the photodynamic-therapy-induced unfolded protein response, signaling by BRAF and RAF fusions, and antigen processing and presentation (Figure 4). 

### 3.4. circRNA Translation Is Driven by IRES-Like Short Elements

Recent studies have shown that circRNAs have translation potential and generate circRNA-coded peptides [40,43,44,45,46]. Since circRNAs lack a 5′ end, the translation of circRNAs can only be initiated through a cap-independent mechanism that requires an internal ribosome entry site (IRES). Because IRES-like elements are significantly enriched in human circRNAs compared to linear RNAs, suggesting that they are positively selected in circRNAs [40], we queried IRES-like hexamers against the DE circRNA sequences to examine the host genes for IRES binding sites.

**Figure 5:**
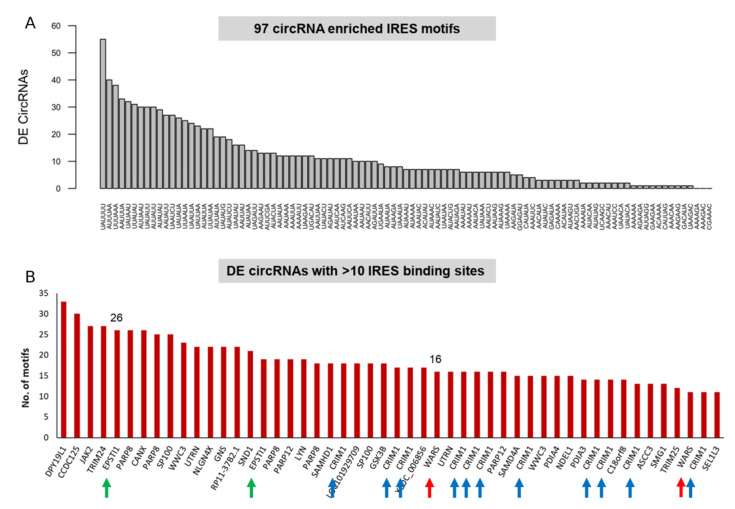
DE circRNAs are enriched in IRES elements. (**A**) The distribution of DE circRNAs that harbor circRNA-enriched IRES motifs. The *x*-axis shows the 96 circRNA-enriched IRES motifs, and the *y*-axis shows the number of DE circRNAs harboring these motifs. (**B**) DE circRNAs harboring more than 10 IRES binding sites, highlighting their protein-coding potential. Arrows indicate the binding sites belonging to the three validated candidates.

Figure 5A shows the occurrence of 97 circRNA-enriched IRES motifs [40] within the DE circRNAs. Most of the DE circRNA candidates harbored an IRES motif within the circRNA sequence, except for four candidates (three of them mapped to the *GRM4* locus, and one mapped to the *NLRC5* locus). We observed that the DE circRNAs were rich in IRES binding sites (95% of DE circRNAs harbored an IRES site). More than 60% of the DE circRNAs (*n* = 53) had >10 IRES binding sites. 

CircRNAs from the *DPY19L1*, *CCDC125, JAK2, TRIM24, EPSTI1, PARP8*, and *CANX* loci had the highest number of IRES motifs within the circRNA sequence (>25 motifs), highlighting their protein-coding potential. Figure 5B shows all the DE circRNAs that harbor >10 IRES motifs. The top candidates, *WARS, EPSTI1,* and *CRIM1,* are highlighted with red, green, and blue arrows, respectively. 

We used the publicly available high-resolution human proteomics data from 30 tissues and six cell lines, as reported in [40], to identify circRNA-coded proteins across the back-splice junctions for all circRNAs expressed in EndoC-βH1 cells. We observed circRNA-coded peptides from 89 β-cell-expressed circRNAs. Two of the circRNAs that encode these short peptides were differentially expressed: hsa_circ_0001565 (circCANX, upregulated) and hsa_circ_0001596 (circHIST1H1B, downregulated) (Supplementary Table S4).

Leveraging the proteomics data retrieved from [29] on EndoC-βH1 cells exposed to IL-1β and IFN-γ for 48 h, we identified peptides matching the potentially translated proteins from the DE circRNAs. The 118,143 peptides from the proteomics data were compared with the ORFs predicted for the DE circRNAs, as detailed in Section 2, to identify identical circRNA-specific sequences. Supplementary Table S5 shows these circ-encoded peptides in EndoC-βH1 cells and their statistical analysis. 

### 3.5. Multi-Species Conserved Sequences (MCS) Analysis

To speculate on the functional relevance of the DE circRNAs, we next investigated the evolutionary conservation of their specific sequences, including their unique junction-spanning signature, using the CircAtlas database [41] in seven species: human (hsa), macaque (mma), mouse (mmu), rat (rno), pig (sus), chicken (gal), and dog (can). Around 70% of the DE circRNAs were conserved in at least two species, and ~15% of the DE circRNAs were conserved in five species (Supplementary Tables S3 and S6). Interestingly, all the DE circRNAs with multi-species conserved sequences (MCS) in five species harbored >10 IRES binding sites (Supplementary Table S3). 

### 3.6. Cytokine-Induced Regulatory Elements in circRNAs

**Figure 6:**
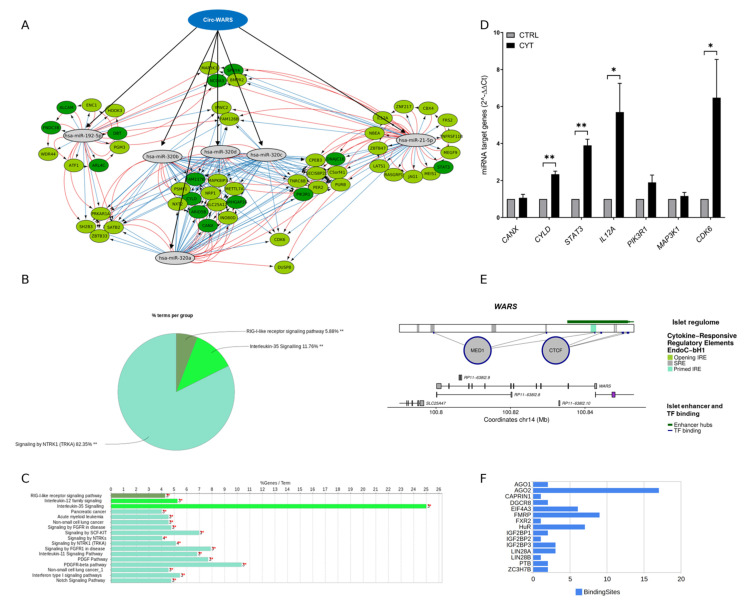
circWARS–miRNA–mRNA interaction network. (**A**) The figure shows the circWARS–miRNA–mRNA network for β-cell-enriched miRNAs (shown as grey nodes): the hsa-miR-192-5p, hsa-miR-21-5p, and hsa-miR-320 family are predicted to bind circWARS. The target genes for the miRNAs were selected based on Targetscan (red edges) and MirTarBase (blue edges). Only those targets that were upregulated by pro-inflammatory cytokines either at the mRNA level (light green nodes) or both the mRNA and protein levels (dark green nodes) in EndoC-βH1 cells are shown; (**B**) Pathway-based enrichment analysis of 54 upregulated target genes of β-cell-enriched miRNAs (hsa-miR-192-5p, hsa-miR-21-5p, and hsa-miR-320 family) with potential binding sites for circWARS revealed three clusters of enriched pathways; (**C**) The individual enriched pathway terms are shown as a bar plot and are clustered into three groups based on color; (**D**) The expression of hsa-miR-192-5p, hsa-miR-21-5p, and the hsa-miR-320 family target genes, *CANX*, *CYLD*, *STAT3*, *IL12A*, *PIK3R1*, *MAP3K1,* and *CDK6* is shown at mRNA level in the EndoC-βH1 cells that were untreated (CTRL) or treated with IL-1β and IFN-γ (CYT) for 48 h (*n* = 3). The normalized expression values are presented as 2^−ΔΔCt. *GAPDH* was used as a housekeeping gene. * *p* < 0.05, ** *p* < 0.01. (**E**) Promoter regions of WARS locus harbor islet enhancer hub, islet TF binding sites, and primed cytokine-responsive elements; (**F**) RNA-binding protein (RBP) sites predicted for circWARS.

Leveraging the recently published genome-wide map of inflammation-induced responsive elements in human β cells that are linked to changes in the β-cell transcriptome, proteome, and 3D chromatin structure [29], we identified circRNAs that harbor cytokine-induced primed regulatory elements prebound by islet-specific transcription factors. Of the 84 DE circRNAs, 23 circRNAs overlapped with regulatory elements (REs) (10 opening induced regulatory elements, “open IRE elements”, and 66 stable regulatory elements, “SRE elements”) in EndoC-βH1 cells. In human islets, 41 DE circRNAs overlapped with 3 opening IRE and 199 SRE elements. Interestingly, the promoter regions of our top candidates, *WARS, EPSTI1,* and *CRIM1,* harbored islet enhancer hubs, islet TF binding sites, and primed cytokine-responsive elements (Figure 6 and Supplementary Figure S6). The circRNAs from these loci that harbored SREs included: circCRIM1 and circEPSTI1 in EndoC-βH1 cells and circWARS and circCRIM1 in human islets. Interestingly, out of the 17 DE circRNA isoforms at the *CRIM1* locus, 13 harbored > 100 SREs (Supplementary Figure S6).

### 3.7. RNA-Binding Protein (RBP) and miRNA Binding Sites within circWARS, circEPSTI1, and circCRIM1

circRNAs with a relatively high density of binding sites for any single RBP could potentially act as a ‘sponge’ or a ‘decoy’ for that RBP [15]. This sponging function would be enhanced by the long half-lives of circRNAs. Therefore, we used the CircInteractome database [38] to predict the RBP binding sites for the three validated circRNAs (circWARS, circEPSTI1, and circCRIM1). Figure 6 and Supplementary Figure S5 show the total number of binding sites for RBPs (e.g., Ago1, Ago2, Hur, FMRP, and EIF4A3) overlapping the junction sequence of circWARS, circEPSTI1, and circCRIM1. Multiple binding sites for AGO1 and AGO2 on circWARS and circCRIM1 were also confirmed by the StarBase analysis (Supplementary Table S7). CircRNAs with a high density of binding sites for a given RBP might be considered ‘super-sponges’ [14,15]. Therefore, we speculate that hsa_circ_0033184 (circWARS, 626 nt long) could function as a super-sponge for AGO2 in pancreatic β cells. 

The circRNA–miRNA interactions are mediated by the RNA-induced silencing complex (RISC) containing AGO2 and many other RNA-binding proteins. Different isoforms of circWARS-interacting miRNAs were identified by intersecting the predicting target sites of miRNAs with the binding sites of the Ago protein derived from publicly available CLIP-seq data from the StarBase database [32] (Supplementary Table S7). The miRNAs predominantly expressed in isolated β cells compared to human islets were identified in [37].

For the most highly upregulated circRNAs, circWARS, we identified binding sites for 29 miRNAs, of which 14 were β-cell-enriched compared to human islets (Supplementary Table S7). hsa-miR-192-5p, one of the most highly β-cell-enriched miRNAs, previously reported by Klein et al. [47], was supported by >15 Ago Clip-Seq experiments, underlining the circWARS–hsa-miR-192-5p interaction. The other β-cell-enriched miRNAs targeted by circWARS included hsa-miR-320a, hsa-miR-320b, hsa-miR-320c, hsa-miR-320d, and hsa-miR-21-5p. Our analysis did not identify any miRNA binding sites for circEPSTI1. For the 16 DE circRNAs from the *CRIM1* locus, binding sites for more than 300 miRNAs were identified (data not shown). Interestingly, both circCRIM1 and circWARS have binding sites for highly β-cell-enriched miRNAs, including hsa-miR-320a, hsa-miR-320b, hsa-miR-320c, hsa-miR-320d, and hsa-miR-4429. 

### 3.8. circWARS–miRNA–mRNA Interaction Network in EndoC-βH1 Cells

A “circWARS-miRNA-mRNA” interaction network was generated by combining the CLIP-seq data from StarBase with the host gene expression (RNASeq) and proteomics data in EndoC-βH1 cells. Figure 6A shows the “circWARS-miRNA-mRNA” interaction network for the six β-cell-enriched miRNAs (hsa-miR-192-5p, hsa-miR-21-5p, hsa-miR-320a, hsa-miR-320b, hsa-miR-320c, and hsa-miR-320d) and their 54 target genes that were upregulated by IL-1β and IFN-γ at 48 h in EndoC-βH1 cells. Only the targets that were upregulated by pro-inflammatory cytokines either at the mRNA level (light green nodes) or both the mRNA and protein level (dark green nodes) in EndoC-βH1 cells were included in the network. Several shared target genes for hsa-miR-320a, hsa-miR-320b, hsa-miR-320c, and hsa-miR-320d were upregulated at both the mRNA and protein levels. These targets included *CANX, CYLD, ARID5B, FAM117B*, and *ARHGAP26*.

A pathway-based enrichment analysis of the 54 upregulated target genes of β-cell-enriched miRNAs (hsa-miR-192-5p, hsa-miR-21-5p, and hsa-miR-320 family) with potential binding sites for circWARS revealed three clusters of enriched pathways (Figure 6B,C). The most significant pathways for these three clusters were: NTRK1 signaling, interleukin-35 signaling, and RIG-I-like receptor signaling. The associated genes for the three enriched clusters included *CYLD, IL12A, MAP3K1* (RIG-I-like receptor signaling), *CANX, IL12A, STAT3* (interleukin-35 signaling), *CDK6, PIK3R1, STAT3, PER2, FRS2, SH2B3, ATF1, MAP3K1,* and *JAG1* (NTRK1 signaling). Real-time qPCR validated the expression of the target genes, *CANX*, *CYLD*, *STAT3*, *IL12A*, *PIK3R1*, *MAP3K1,* and *CDK6,* in the EndoC-βH1 cells with and without treatment with pro-inflammatory cytokines for 48 h (Figure 6D). The mRNA levels of *CYLD*, *STAT3*, *IL12A,* and *CDK6* were significantly upregulated in response to cytokine treatment. The most significantly upregulated target genes, *CYLD* and *STAT3,* were validated in human pancreatic islets with and without cytokine treatment for 24 h (Figure S7A). Both were significantly upregulated in response to cytokine treatment. Differential expression of the miRNAs does not serve as a prerequisite for the circRNA sponge function, and with real-time qPCR we did not observe significant changes in the miRNA levels. However, we were able to validate the expression of hsa-miR-192-5p, hsa-miR-21-5p, hsa-miR-320a, and hsa-miR-320b in human islets with and without cytokine treatment (Figure S7B). 

Figure 6E shows the genomic map of the *WARS* locus and the overlap with IREs, islet enhancer hubs, and islet TF binding sites. Multiple SREs and primed IREs were present in the circWARS sequence based on the islet regulome data. Interestingly, for circWARS, more than 15 AGO2 binding sites were observed, along with multiple binding sites for other RBPs, including *FMRP, HuR, EIF4A3, IGF2BP1, IGFBP2,* and *IGFBP3* (Figure 6F and Supplementary Table S7). 

## 4. Discussion

In the present study, we investigated the circRNA expression profile in human β cells exposed to pro-inflammatory cytokines and identified their potential roles in T1D. Using microarray profiling, we detected more than 10,000 circRNAs mapping to more than 5000 genes in the EndoC-βH1 cells. After 24 h of pro-inflammatory cytokine exposure, 74 circRNAs were differentially expressed (71 significantly upregulated and 3 significantly downregulated), and after 48 h of pro-inflammatory cytokine exposure, 50 circRNAs were differentially expressed (48 significantly upregulated and 2 significantly downregulated). Interestingly, the host genes of the DE circRNAs were found to be implicated in interferon and interleukin signaling, which suggests that the DE circRNAs are transcribed from inflammatory response genes in response to a pro-inflammatory stimulus.

The cell-type-specific expression of circRNAs in human pancreatic islets makes them attractive as potential targets for new therapeutic strategies to rescue and/or improve β-cell function in diabetes [48,49,50]. For instance, Stoll et al. reported that a β-cell circRNA derived from the insulin gene was found to be reduced in islets from individuals with type 2 diabetes (T2D) and in rodent diabetes models. This circRNA was found to regulate insulin secretion, as silencing the circRNA impaired insulin secretion and key insulin signaling pathways [49]. Furthermore, a reduction in the level of one of the most abundant islet circRNAs, circHIPK3, has been shown to promote apoptosis and reduce the proliferation of β cells in mice [17,25]. The silencing of another abundantly expressed circRNA, circAFF1, in pancreatic islets of human, mouse, and rat as well as in insulin-secreting INS832/13 and MIN6B1 cell lines induced apoptosis after treatment with pro-inflammatory cytokines [17]. These examples suggest that strategies to increase the levels of certain circRNAs may hold the potential to prevent apoptosis and ameliorate β-cell mass and function. More functional studies, however, are warranted to explore the full potential of circRNAs as novel disease-modulating targets in human β cells.

The effects of cytokines on circRNAs in β cells have so far been studied only in mouse models [23]. Using a microarray analysis, Wang et al. reported close to 2000 DE circRNAs in MIN6 cells after 24 h of IL-1β, IFN-γ, and TNFα treatment [23]. In contrast to the present study, MIN6 cells were found to express a higher number of circRNAs in general and contained approximately equal amounts of up- and downregulated candidates [23]. This could reflect interspecies differences between human and mouse. By siRNA-mediated knockdown, the group inhibited two DE circRNAs, which resulted in elevated β-apoptosis as well as reduced insulin biosynthesis and secretion [23]. Others have described differences in the circRNA expression profile, primarily in PBMCs from individuals with T1D, compared to healthy controls [20,21,22], further supporting their roles in islet inflammation and β-cell destruction. To our knowledge, only two other studies have investigated circRNAs in isolated human pancreatic islets [17,25]. Using CircleSeq, Haque et al. recently identified 2619 circRNAs in human islets and reported by real-time qPCR that four of the most abundantly expressed circRNAs were differentially expressed when comparing islets of individuals with and without T2D [25]. By microarray analysis, Stoll et al. identified 3441 circRNA transcripts in human islets in baseline conditions, of which they showed that 497 were conserved in mouse islets [17]. Selected circRNA candidates were validated in mouse islets and investigated for their functional roles by siRNA-mediated knockdown. The study observed an impairment of insulin secretion as well as reduced proliferation and survival of the mouse islets [17]. The evolutionary conservation of circRNA candidates reported in the study by Stoll et al. has also been discussed by others [13] and supports the current study’s findings. Thus, the circRNA sequences seem to be functionally conserved, and the differential expression of these in response to diabetogenic factors implies their potential functionalities and possible implications in T1D pathogenesis [17,24]. 

We selected three of the most highly and DE circRNAs, circWARS (hsa_circ_0033184), circEPSTI1 (hsa_circ_0000479), and circCRIM1 (hsa_circ_0002938), for further studies and validated their presence and circular nature by Sanger sequencing. We highlight the protein-coding potential of circWARS, circCRIM1, and circEPSTI1, showing that they, together with other DE circRNAs, were rich in IRES elements. The translational potential of a subset of circRNAs has been described in recent years, suggesting that the circRNAs are prone to translation through the cap-independent binding of ribosomes to IRES [40,51]. A novel study by Fan et al. showed that sufficient levels of IRES elements were able to drive the translation of circRNAs, identifying hundreds of circRNA peptides [40]. Furthermore, they also showed that the mutation of the IRES-like short elements in circRNAs reduced their translation. Other studies have shown that RNA modifications, including a single-nucleotide N6-methyladenosine (m6A) modification on circRNAs, are sufficient to promote translation via the recruitment of initiation factor eukaryotic translation factor 4 gamma (elF4G2) and m6A reader, YTHDF [52,53]. The circRNA-encoded peptides identified in this study should be validated by other methods since we cannot be sure from which protein these peptides were generated during the sample digestion that preceded the mass spectrometry analysis. Moreover, future studies are warranted to address the biological relevance of the circRNA-derived peptides.

The most highly upregulated candidate of this study, circWARS, has to our knowledge not previously been described in the literature. However, its host gene, *WARS,* encodes the enzyme tryptophanyl-tRNA synthetase, which is known to catalyze the tryptophan-tRNA ligation, which is key for protein synthesis [54]. *WARS* has been previously studied for its role in the innate inflammatory response, where it was found to act via Toll-like receptor activation and regulate the immune cell response [54,55,56]. In the present study, we showed that circWARS harbored multiple binding sites for key β-cell miRNAs and RBPs, including Ago2. Thus, a potential role of circWARS could be to sponge miRNAs, preventing them from reaching their targets, hence affecting downstream molecular pathways. The ‘circWARS-miRNA-mRNA’ network analysis revealed multiple binding sites for hsa-miR-192-5p, hsa-miR-21-5p, and several members of the miR-320 family, which is known to be highly expressed in human pancreatic islets and β cells [37]. The miR-320 miRNA family has been studied for its role in diabetes and related complications, particularly for its role in glucose and lipid metabolism [57,58,59,60]. A recent study showed that the overexpression of miR-320a suppressed insulin secretion and induced apoptosis in pancreatic β cells via targeting MafF in high fat diet treated mice [58]. Consistent with an miRNA sponging effect of circWARS, a number of target genes for hsa-miR-320a, hsa-miR-320b, hsa-miR-320c, and hsa-miR-320d were upregulated, including *CANX, CYLD, ARID5B, FAM117B,* and *ARHGAP26*. *CANX* encodes an endoplasmic reticulum (ER) membrane chaperone, calnexin, which plays an important role in the unfolded protein response and ER-associated degradation (ERAD) [61]. Indeed, calnexin-deficient cells upregulate the ER-stress-associated protein Erp57 and show both increased proteasomal activity and increased ERAD [62]. The upregulated shared target gene *PIK3R1* for hsa-miR-21-5p, hsa-miR-320a, hsa-miR-320b, hsa-miR-320c, and hsa-miR-320d was upregulated at both the mRNA and protein levels, as shown by the ‘circWARS-miRNA-mRNA’ network analysis. Several studies in transgenic mouse models have shown that the inhibition of Pik3r1 enhances *PI3K* activity and improves insulin signaling and glucose homeostasis in high fat diet fed obese mice and mice with genetically induced insulin resistance [63]. circWARS also harbored multiple binding sites for *IGFBP1* and *IGFBP2*. *IGFBP1* has previously been linked to diabetes/insulin regulation, as lower circulating levels of *IGFBP1* have been associated with impaired glucose tolerance, insulin resistance, diabetes, and cardiovascular diseases in mouse models [64]. Thus, we speculate that the regulation of the binding targets of circWARS could mediate important effects for β-cell function. The current study does not investigate the binding effects of circWARS. However, according to real-time qPCR, several of the miRNA target genes were significantly upregulated in response to cytokine treatment. Future studies should address the binding and regulation of circWARS to its miRNA and protein targets to establish the biological mechanism of this circRNA. 

circCRIM1 was implicated in promoting ovarian cancer progression by acting as a competitive endogenous RNA (ceRNA) for the host gene *CRIM1* and targeting the miR-383-5p/ZEB2 axis [65]. *CRIM1* encodes a transmembrane protein containing six cysteine-rich repeat domains and an insulin-like growth-factor-binding domain. It has been shown to play a role in tissue development through its interaction with the transforming growth factor beta (TGFβ) family and bone morphogenetic proteins (BMP) [66,67]. circEPSTI1 has been proposed as a biomarker of systemic lupus erythematosus (SLE), and significantly elevated levels of circEPSSTI1 were found in PBMCs from individuals with SLE compared to healthy controls [68]. The host gene, *EPSTI1,* encodes the epithelial–stromal interaction 1 protein, which has been studied for its role in cancer [69].

Due to the circular nature of circRNAs, these molecules are more resistant to exonuclease breakdown than their linear counterparts, and therefore circRNAs may be ideal biomarkers [5,70]. Although circRNAs are often sparsely expressed compared to their host genes, their biological properties and individual functions seem to have profound roles in disease [5,70,71]. However, the distinct functionalities of the circular and linear transcripts should be clarified to ensure that the effects arise from the circRNA and not the linear counterparts. One of the limitations of the present study is that the speculated circRNA functions rely on literature based on the host genes. Moreover, the lack of proper annotations of the circRNAs is an issue for the pathway analysis, where all genes were used as a background instead of only those associated with the circRNAs. We observed that the host genes of the three validated circRNA candidates were also differentially expressed in the same direction as the circRNAs. This could be a confounding factor of the speculated functionalities of the circRNAs, highlighting the importance of separating the linear and circular transcripts when investigating circRNAs’ modes of action. However, other studies have reported differential expression of circRNAs in both the same and opposite directions as their host gene [17,22,25,49]. This suggests that circRNAs and host genes may be coregulated or even modulate the expression of each other [72], underlining our still limited understanding of the complex nature of circRNAs. Further studies are needed to determine the ultimate functions of circRNAs and their eventual clinical relevance and utility as biomarkers.

In conclusion, we show that circRNAs are highly abundant in human pancreatic β cells and that they are differentially expressed in response to pro-inflammatory cytokines. Based on these findings, we predict that the DE circRNAs are functionally conserved and may be involved in regulating β-cell function. Examining the molecular mechanisms of the individual β-cell circRNAs should be the subject of future investigations to elucidate their implication in β-cell loss and dysfunction in T1D.

## Figures and Tables

**Table 1:** Upregulated circRNAs after cytokine exposure at both 24 h and 48 h in human EndoC-βH1 cells. The table shows 40 circRNAs upregulated at both 24 and 48 h. The circRNA array IDs and the circBase IDs (wherever available) are shown for all candidates, along with their log fold changes and adjusted *p*-values.

circRNA			24 h		48 h	
Gene	circRNA	circBase ID	logFC	adj. *p*-Value	logFC	adj. *p* Value
*WARS*	hsa_circRNA_101439	hsa_circ_0033184	4.72	4.7 × 10^−8^	3.87	5.0 × 10^−7^
*WARS*	hsa_circRNA_033191	hsa_circ_0033191	3.18	1.4 × 10^−3^	2.99	1.1 × 10^−3^
*EPSTI1*	hsa_circRNA_000479	hsa_circ_0000479	3.55	1.0 × 10^−5^	3.59	9.0 × 10^−6^
*EPSTI1*	hsa_circRNA_405138		1.67	5.5 × 10^−5^	1.91	1.4 × 10^−5^
*IRF1*	hsa_circRNA_001526	hsa_circ_0001526	3.37	1.1 × 10^−7^	2.99	5.0 × 10^−7^
*UTRN*	hsa_circRNA_001646	hsa_circ_0001646	2.23	1.5 × 10^−5^	1.96	5.4 × 10^−5^
*UTRN*	hsa_circRNA_001648	hsa_circ_0001648	2.17	1.4 × 10^−3^	1.84	2.9 × 10^−3^
*CRIM1*	hsa_circRNA_102689	hsa_circ_0054021	1.76	1.5 × 10^−3^	1.38	6.8 × 10^−3^
*CRIM1*	hsa_circRNA_102686	hsa_circ_0053967	1.82	2.2 × 10^−3^	1.44	9.8 × 10^−3^
*CRIM1*	hsa_circRNA_007408	hsa_circ_0007408	1.62	2.3 × 10^−3^	1.27	1.0 × 10^−2^
*CRIM1*	hsa_circRNA_102688	hsa_circ_0006422	1.64	4.5 × 10^−3^	1.17	3.5 × 10^−2^
*CRIM1*	hsa_circRNA_102687	hsa_circ_0002938	2.02	6.9 × 10^−3^	1.58	3.1 × 10^−2^
*CRIM1*	hsa_circRNA_102681	hsa_circ_0053958	1.87	8.2 × 10^−3^	1.84	1.0 × 10^−2^
*CRIM1*	hsa_circRNA_005507	hsa_circ_0005507	1.62	9.2 × 10^−3^	1.74	6.8 × 10^−3^
*CRIM1*	hsa_circRNA_405851		1.42	1.2 × 10^−2^	1.49	1.0 × 10^−2^
*CRIM1*	hsa_circRNA_102682	hsa_circ_0005442	1.56	1.3 × 10^−2^	1.78	6.8 × 10^−3^
*CRIM1*	hsa_circRNA_102679	hsa_circ_0005579	1.55	1.3 × 10^−2^	1.43	2.5 × 10^−2^
*CRIM1*	hsa_circRNA_102677	hsa_circ_0002017	1.44	1.3 × 10^−2^	1.75	5.4 × 10^−3^
*CRIM1*	hsa_circRNA_053955	hsa_circ_0053955	1.61	1.4 × 10^−2^	1.91	6.8 × 10^−3^
*CRIM1*	hsa_circRNA_102680	hsa_circ_0002348	1.74	2.0 × 10^−2^	1.79	2.3 × 10^−2^
*CRIM1*	hsa_circRNA_102678	hsa_circ_0002346	1.57	2.1 × 10^−2^	1.82	1.0 × 10^−2^
*CRIM1*	hsa_circRNA_004182	hsa_circ_0004182	1.55	2.1 × 10^−2^	1.7	1.5 × 10^−2^
*CRIM1*	hsa_circRNA_006294	hsa_circ_0006294	1.78	2.2 × 10^−2^	1.77	3.0 × 10^−2^
*CRIM1*	hsa_circRNA_102685	hsa_circ_0003578	1.61	3.2 × 10^−2^	1.69	3.0 × 10^−2^
*IFI30*	hsa_circRNA_102484	hsa_circ_0005571	1.92	1.4 × 10^−3^	2.01	7.0 × 10^−4^
*STAT3*	hsa_circRNA_401803		1.77	4.9 × 10^−3^	1.36	2.5 × 10^−2^
*STAT3*	hsa_circRNA_102073	hsa_circ_0043812	1.31	1.4 × 10^−3^	1.28	1.1 × 10^−3^
*GRM4*	hsa_circRNA_104092	hsa_circ_0076041	1.44	7.9 × 10^−3^	1.59	5.4 × 10^−3^
*GRM4*	hsa_circRNA_104091	hsa_circ_0076040	1.31	2.8 × 10^−2^	1.57	1.2 × 10^−2^
*GRM4*	hsa_circRNA_076039	hsa_circ_0076039	1.32	1.1 × 10^−2^	1.46	6.8 × 10^−3^
*SAMD4A*	hsa_circRNA_101356	hsa_circ_0004846	1.63	1.4 × 10^−3^	1.71	7.0 × 10^−4^
*SAMHD1*	hsa_circRNA_406106		1.6	1.7 × 10^−3^	1.07	2.3 × 10^−2^
*LOC101928767*	hsa_circRNA_405273		1.61	4.0 × 10^−3^	1.4	9.8 × 10^−3^
*LOC101929709*	hsa_circRNA_002576	hsa_circ_0002576	1.21	5.6 × 10^−3^	1.18	6.8 × 10^−3^
*PARP12*	hsa_circRNA_082689	hsa_circ_0082689	1.39	1.3 × 10^−2^	1.32	2.3 × 10^−2^
*PARP8*	hsa_circRNA_103835	hsa_circ_0072431	1.35	5.8 × 10^−3^	1.04	3.0 × 10^−2^
*DENND3*	hsa_circRNA_407136		1.3	3.2 × 10^−3^	1.01	1.5 × 10^−2^
*FRAS1*	hsa_circRNA_103671	hsa_circ_0070098	1.2	5.8 × 10^−3^	1.63	6.9 × 10^−4^
*NLRC5*	hsa_circRNA_101819	hsa_circ_0039522	1.08	1.7 × 10^−3^	1.37	2.6 × 10^−4^
*XLOC_006856*	hsa_circRNA_001200	hsa_circ_0001812	1.16	8.1 × 10^−3^	1.2	7.6 × 10^−3^

## Data Availability

The circRNA microarray data from this study were submitted to the GEO database (accession number: GSE209612).

## Supplementary Materials

The following supporting information can be downloaded at: https://www.mdpi.com/article/10.3390/ncrna8050069/s1, Table S1: Pancreatic islet donor information; Table S2: Genomic characteristics of 84 DE circRNAs; Table S3: Primer sequences for circRNAs; Table S4: Protein-coding potential of DE circRNAs based on published studies; Table S5: Differentially expressed circ-encoded peptides in EndoC-βh1 cells; Table S6: Multi-species conserved sequences (MCS) analysis of the 84 DE circRNAs; Table S7: Predicted interactions between the two differentially expressed circWARS isoforms and miRNAs; Figure S1: Verification of circWARS, circEPSTI1, and circCRIM1 expression in EndoC-βH1; Figure S2: Verification of circWARS and circEPSTI1 expression in human islets; Figure S3: Sanger sequencing confirmed the expression of circWARS, circEPSTI1, and circCRIM1 in EndoC-βH1 cells; Figure S4: The expression of host genes *WARS*, *EPSTI1*, and *CRIM1* in human islets; Figure S5: The total number of RNA-binding protein (RBP) binding sites on junction sequences of top DE circRNAs (circWARS, circEPSTI1, and circCRIM1); Figure S6: *CRIM1* and *EPSTI1* loci harbor multiple cytokine-responsive elements (islet regulome); Figure S7: The expression of miRNA targets and miRNA levels in human islets.
