# Supplementary material for: Pro-Inflammatory Cytokines Promote the Transcription of Circular RNAs in Human Pancreatic β Cells
Source: Noncoding RNA. 2022 Oct 12;8(5):69. doi: 10.3390/ncrna8050069 (PMC9609391; doi:10.3390/ncrna8050069)
Supplement: Supplementary file 1 [file ncrna-08-00069-s001.zip › ncrna-1907350-supplementary.pdf]

Supplementary Tables

Table S1. Pancreatic islet donor information: unique identifier, gender: male (M)/ female (F), age (years), BMI (kg/m²), HbA1c (%), origin/source, isolation centre, diabetes history, cause of death. islet viability (%) and purity (%).

**Unique identifier**	**HP-18270-01**	**HP-18304-01**	**HP-18310-01**	**HP-18341-01**
**Gender (M/F)**	M	M	M	M
**Donor age (years)**	31	21	38	48
**BMI (kg/m²)**	22.0	27.3	28.0	26.6
**HbA1c (%)**	5.5	5.4	5.9	5.1
**Origin/source**	UNOS	UNOS	UNOS	UNOS
**Isolation centre**	Prodo Labs	Prodo Labs	Prodo Labs	Prodo Labs
**Diabetes history**	No	No	No	No
**Cause of death**	Anoxic event	Head trauma	Head trauma	Stroke
**Viability (%)**	95	95	95	95
**Purity (%)**	90-95	85	90	90

Table S2. Primer sequences for circRNAs.

Primer	Left Primer	Right Primer
hsa_circ_0033184 (circWARS)	CTGTGGAGAATGCCAAGGAC	GTTCCCTGGAGGACAGTCAG
hsa_circ_0000479 (circEPSTI1)	GGCAATTCAGAGAGAGAAGAGC	CCTTCCACTTCTCCAGGTTG
hsa_circ_0002938 (circCRIM1)	TCTGAGTCCTGCCCTTCTGT	GTCCTTCCCTGTCAGAGTGC

Table S3. Genomic characteristics of the 84 DE circRNAs**.**

The table lists all the 84 DE circRNAs along with their probe IDs (Arraystar ID), circBase ID, their genomic coordinates (hg38), associated host gene name (Gene Symbol), the differential expression status (DE status), total number of IRES binding sites, circRNAs biotype, the number of species the circRNAs sequence is conserved in, and the spliced length of the circRNAs.

Arraystar ID	circBase_ID	hg38	Gene Symbol	DE status	Total IRES binding motifs	circRNA type	Multiple conserved species (MCS)	Spliced length
hsa_circRNA_000479	hsa_circ_0000479	chr13:42953948|42970670	EPSTI1	up	26	exon	2	499
hsa_circRNA_000787	hsa_circ_0000787	chr17:56901419|56904488	TRIM25	up	12	exon	2	394
hsa_circRNA_000897	hsa_circ_0000897	chr19:12943767|12944036	CALR	up	4	Non-Repeat	1	192
hsa_circRNA_001059	hsa_circ_0000554	chr14:74084975|74085256	LIN52	down	4	sense overlapping	NA	282
hsa_circRNA_001200	hsa_circ_0001812	chr8:78933743|78943071	XLOC_006856	up	17	intergenic	1	458
hsa_circRNA_001496	hsa_circ_0001496	chr5:69294793|69314043	CCDC125	up	30	exon	1	534
hsa_circRNA_001526	hsa_circ_0001526	chr5:132487039|132488022	IRF1	up	5	Non-Repeat	1	186
hsa_circRNA_001565	hsa_circ_0001565	chr5:179709873|179716294	CANX	up	26	exon	3	383
hsa_circRNA_001596	hsa_circ_0001596	chr6:27867023|27867106	HIST1H1B	down	4	sense overlapping	NA	84
hsa_circRNA_001646	hsa_circ_0001646	chr6:144521980|144533260	UTRN	up	16	exon	2	368
hsa_circRNA_001648	hsa_circ_0001648	chr6:144781922|144803147	UTRN	up	22	exon	3	481
hsa_circRNA_001766	hsa_circ_0001766	chr7:149018992|149021147	PDIA4	up	15	exon	2	387
hsa_circRNA_001803	hsa_circ_0001803	chr8:55941855|55953984	LYN	up	19	exon	6	795
hsa_circRNA_002084	hsa_circ_0002084	chr19:48913011|48913564	NUCB1	up	7	exon	3	277
hsa_circRNA_002576	hsa_circ_0002576	chr8:89724629|89725641	LOC101929709	up	18	exon	1	462
hsa_circRNA_002757	hsa_circ_0002757	chr18:23507985|23519174	C18orf8	up	14	exon	2	163
hsa_circRNA_002824	hsa_circ_0002824	chr9:5021963|5044520	JAK2	up	27	exon	5	493
hsa_circRNA_004182	hsa_circ_0004182	chr2:36369108|36396787	CRIM1	up	2	intron	1	316
hsa_circRNA_004541	hsa_circ_0004541	chr16:58675940|58676346	SLC38A7 (down)	down	1	exon	2	407

hsa_circRNA_005507	hsa_circ_0005507	chr2:36378357|36396787	CRIM1	up	4	exon	1	343
hsa_circRNA_005809	hsa_circ_0005809	chr5:50750151|50763242	PARP8	up	26	exon	6	469
hsa_circRNA_006294	hsa_circ_0006294	chr2:36358904|36396787	CRIM1	up	2	intron	1	401
hsa_circRNA_006497	hsa_circ_0006497	chr3:120002046|120002239	GSK3B (down)	down	18	exon	4	194
hsa_circRNA_007250	hsa_circ_0007250	chr2:10791795|10793202	PDIA6	up	7	exon	2	238
hsa_circRNA_007408	hsa_circ_0007408	chr2:36537462|36537546	CRIM1	up	1	exon	1	195
hsa_circRNA_018818	hsa_circ_0018818	chr10:73504850|73505426	USP54 (down)	down	3	exon	1	260
hsa_circRNA_033191	hsa_circ_0033191	chr14:100360554|100361921	WARS	up	11	exon	4	323
hsa_circRNA_053955	hsa_circ_0053955	chr2:36396614|36499347	CRIM1	up	15	exon	4	1170
hsa_circRNA_057360	hsa_circ_0057360	chr2:188985665|188994332	COL3A1	up	2	exonic	NA	960
hsa_circRNA_058619	hsa_circ_0058619	chr2:230450172|230464150	SP100	up	25	exonic	NA	405
hsa_circRNA_076039	hsa_circ_0076039	chr6:34056544|34133859	GRM4	up	-	exon	2	432
hsa_circRNA_082688	hsa_circ_0082688	chr7:140046884|140058034	PARP12	up	19	exon	4	660
hsa_circRNA_082689	hsa_circ_0082689	chr7:140054662|140058034	PARP12	up	16	exon	3	536
hsa_circRNA_089866	hsa_circ_0089866	chrX:10063445|10110020	WWC3	up	15	exon	2	353
hsa_circRNA_092448	hsa_circ_0000811	chr17:80325030|80337997	RNF213	up	8	exon	2	649
hsa_circRNA_100549	hsa_circ_0004630	chr10:12081472|12094271	DHTKD1	up	9	exon	2	355
hsa_circRNA_101356	hsa_circ_0004846	chr14:54702062|54702580	SAMD4A	up	15	exon	7	519
hsa_circRNA_101439	hsa_circ_0033184	chr14:100353687|100361921	WARS	up	16	exon	4	397
hsa_circRNA_101728	hsa_circ_0006735	chr16:16251963|16252522	NOMO3	up	1	exonic	NA	228
hsa_circRNA_101737	hsa_circ_0006434	chr16:18841565|18845651	SMG1	up	13	exon	6	453
hsa_circRNA_101819	hsa_circ_0039522	chr16:57017074|57021007	NLRC5	up	-	exon	2	422
hsa_circRNA_101978	hsa_circ_0005869	chr17:8444260|8460160	NDEL1	up	15	exon	6	404
hsa_circRNA_102073	hsa_circ_0043812	chr17:42324711|42325061	STAT3	up	2	exon	2	351
hsa_circRNA_102074	hsa_circ_0043815	chr17:42345559|42348517	STAT3	up	8	exon	2	373
hsa_circRNA_102075	hsa_circ_0043816	chr17:42345559|42348539	STAT3	up	9	exon	4	395
hsa_circRNA_102484	hsa_circ_0005571	chr19:18175040|18175697	IFI30	up	6	exon	1	351
hsa_circRNA_102677	hsa_circ_0002017	chr2:36396614|36396787	CRIM1	up	16	exon	5	174
hsa_circRNA_102678	hsa_circ_0002346	chr2:36396614|36442735	CRIM1	up	16	exon	2	295
hsa_circRNA_102679	hsa_circ_0005579	chr2:36396614|36464655	CRIM1	up	16	exon	6	660
hsa_circRNA_102680	hsa_circ_0002348	chr2:36396614|36479694	CRIM1	up	17	exon	1	919
hsa_circRNA_102681	hsa_circ_0053958	chr2:36396614|36517542	CRIM1	up	14	exon	2	390
hsa_circRNA_102682	hsa_circ_0005442	chr2:36396614|36522313	CRIM1	up	17	exon	4	874
hsa_circRNA_102685	hsa_circ_0003578	chr2:36441258|36479694	CRIM1	up	13	exon	5	441
hsa_circRNA_102686	hsa_circ_0053967	chr2:36441258|36522313	CRIM1	up	11	exon	6	465
hsa_circRNA_102687	hsa_circ_0002938	chr2:36499219|36522313	CRIM1	up	10	exon	4	508
hsa_circRNA_102688	hsa_circ_0006422	chr2:36509983|36522313	CRIM1	up	10	exon	3	379
hsa_circRNA_102689	hsa_circ_0054021	chr2:36522092|36522313	CRIM1	up	14	exon	1	222
hsa_circRNA_102940	hsa_circ_0003922	chr2:230442937|230450255	SP100	up	18	exon	1	713
hsa_circRNA_103671	hsa_circ_0070098	chr4:78237510|78255375	FRAS1	up	6	exon	2	495
hsa_circRNA_103672	hsa_circ_0070100	chr4:78237510|78286539	FRAS1	up	9	exon	4	243
hsa_circRNA_103674	hsa_circ_0070113	chr4:78265025|78267432	FRAS1	up	5	exon	1	378
hsa_circRNA_103834	hsa_circ_0072430	chr5:50750151|50778650	PARP8	up	25	exon	6	129
hsa_circRNA_103835	hsa_circ_0072431	chr5:50750151|50797233	PARP8	up	19	exon	3	134
hsa_circRNA_103836	hsa_circ_0072437	chr5:50778069|50797233	PARP8	up	18	exon	4	157
hsa_circRNA_104032	hsa_circ_0075303	chr5:179719668|179719781	CANX	up	3	exon	2	241
hsa_circRNA_104091	hsa_circ_0076040	chr6:34091883|34133859	GRM4	up	-	exon	3	1180
hsa_circRNA_104092	hsa_circ_0076041	chr6:34132978|34133859	GRM4	up	-	5-utr	4	882
hsa_circRNA_104160	hsa_circ_0077495	chr6:100798713|100805880	ASCC3	up	13	exon	4	594
hsa_circRNA_104470	hsa_circ_0082141	chr7:127807484|127904819	SND1	up	21	exon	4	375
hsa_circRNA_104502	hsa_circ_0082580	chr7:138519189|138551180	TRIM24	up	27	exon	1	483
hsa_circRNA_104629	hsa_circ_0084552	chr8:55966715|55969793	LYN	up	7	exon	1	256
hsa_circRNA_104979	hsa_circ_0007685	chrX:10063445|10067490	WWC3	up	23	exon	5	314
hsa_circRNA_400983	-	chr12:64728956|64744907	GNS	up	22	exon	2	452
hsa_circRNA_401803	-	chr17:42324711|42329647	STAT3	up	8	exon	1	230
hsa_circRNA_403102	-	chr4:25847294|25847864	SEL1L3	up	11	exon	4	571
hsa_circRNA_403802	-	chr7:35010468|35018596	DPY19L1	up	33	exon	6	466
hsa_circRNA_404313	-	chrX:6029280|6032750	NLGN4X	up	22	exon	2	213
hsa_circRNA_405138	-	chr13:42926336|42964139	EPSTI1	up	19	exon	1	326
hsa_circRNA_405273	-	chr14:86028369|86041833	LOC101928767	up	6	intron	1	555
hsa_circRNA_405330	-	chr15:43768489|43769646	PDIA3	up	14	exon	4	238
hsa_circRNA_405851	-	chr2:36387972|36396787	CRIM1	up	18	intron	1	278
hsa_circRNA_406106	-	chr20:36897822|36898544	SAMHD1	up	18	exon	2	243
hsa_circRNA_407094	-	chr8:89647740|89648613	RP11-37B2.1	up	22	intron	1	163
hsa_circRNA_407136	-	chr8:141160632|141163429	DENND3	up	10	exon	2	253

Table S4. Protein-coding potential of DE circRNAs based on published studies.

Two circRNA-coded peptides are from two DE candidates: circ-CANX (up-regulated) and circ-HIST1H1B (down-regulated). The data were derived from [28].

CircBAse ID	CHR	Strand	circ start	circ end	Gene	No. of IRES binding sites	Protein sequence	circRNA cell-line
hsa_circ_0001565	chr5	+	179136873	179143295	CANX	26	IPDPEAVKPDDWISSMTR	cd_34, Hepg2, Helas3, H1hesc, Gm12878, Ag04450, HEK293
hsa_circ_0001596	chr6	-	27834800	27834884	HIST1H1B	4	PAAAGVKKAKK	cd_19

Table S5. Differentially expressed circ-encoded peptides in EndoC-βH1 cells

Protein	Pep1	Peptide	Pep2	Spectra	MSGF_SpecProb	p_value	q_value	Log2FC
NP_001002264.1 (EPSTI, hsa-circ-0000479)	R	IAEQELANLEK	W	2	6.11E-14	0.0009	0.0646	1.2907
NP_005073.2 (TRIM25, hsa-circ-0000787	K	ALLDASETTSTR	K	2	2.33E-15	0.0126	0.0927	0.2292
NP_005073.2 (TRIM25, hsa-circ-0000787	K	ALLDASETTSTRK	I	1	2.22E-12	0.0099	0.0885	0.1778
NP_005073.2 (TRIM25, hsa-circ-0000787	K	RDEFEFLEK	A	1	1.54E-11	0.0897	0.1656	0.1781
NP_005073.2 (TRIM25, hsa-circ-0000787	K	SEIQTLKEEIEQSLTK	R	1	1.5E-17	0.6393	0.4151	0.1064
NP_005073.2 (TRIM25, hsa-circ-0000787	R	DEFEFLEK	A	2	2.23E-11	0.058	0.1431	0.2124
NP_005073.2 (TRIM25, hsa-circ-0000787	R	GISTKPVYIPEVELNHK	L	2	2.06E-20	0.0348	0.1213	0.2139
NP_005073.2 (TRIM25, hsa-circ-0000787	R	KVEQLQQEYTEM.*K	A	4	1.12E-17	0.2805	0.2708	0.1118
NP_005073.2 (TRIM25, hsa-circ-0000787	R	KVEQLQQEYTEMK	A	4	5.37E-18	0.0008	0.0653	0.2539
NP_005073.2 (TRIM25, hsa-circ-0000787	R	VNSKFDTIYQILLK	K	2	1.51E-17	0.0422	0.1292	0.2008
NP_001019820.1 (CANX, hsa-circ-0001565)	K	CGEDYKLH	F	1	3.3E-12	0.2647	0.2632	0.1517
NP_001019820.1 (CANX, hsa-circ-0001565)	K	CGEDYKLHF	I	1	1.25E-11	0.0088	0.087	0.3166
NP_001019820.1 (CANX, hsa-circ-0001565)	K	CGEDYKLHFIFR	H	1	7.53E-17	0.0823	0.1605	0.2383
NP_001019820.1 (CANX, hsa-circ-0001565)	K	RPDADLK	T	1	5.74E-10	0.0055	0.0797	0.6105
NP_001019820.1 (CANX, hsa-circ-0001565)	K	TGIYEEK	H	8	4.58E-10	0.0388	0.1256	0.5106
NP_001019820.1 (CANX, hsa-circ-0001565)	K	THLYTLILNPDNSFEILVDQSVVNSGNLLNDMTPPVNPSR	E	1	1.92E-13	#NUM!	#NUM!	#NUM!
NP_001019820.1 (CANX, hsa-circ-0001565)	K	TPELNLDQFHDKTPYTIMFGPDK	C	11	2.48E-30	0.0803	0.1593	0.3092
NP_001019820.1 (CANX, hsa-circ-0001565)	K	TPELNLDQFHDKTPYTIMFGPDKCGEDYK	L	1	8.33E-27	0.0888	0.165	0.2577
NP_001019820.1 (CANX, hsa-circ-0001565)	K	TPYTIMFGPDK	C	4	1.09E-15	0.0346	0.1213	0.2335
NP_001019820.1 (CANX, hsa-circ-0001565)	K	TPYTIMFGPDKCGEDYK	L	3	9.42E-15	0.0064	0.0815	0.2758
NP_001019820.1 (CANX, hsa-circ-0001565)	K	TYFTDK	K	2	8.98E-10	0.0083	0.0861	0.4306
NP_001019820.1 (CANX, hsa-circ-0001565)	K	TYFTDKK	T	4	5.24E-11	0.0065	0.0817	0.4523
NP_001019820.1 (CANX, hsa-circ-0001565)	R	EIEDPEDRKPEDWDERPK	I	7	3.21E-20	0.0068	0.0824	0.3078
NP_001019820.1 (CANX, hsa-circ-0001565)	R	KPEDWDERPK	I	3	4.88E-13	0.2615	0.2617	0.2112
NP_001019820.1 (CANX, hsa-circ-0001565)	Y	TIM.*FGPDKCGEDYK	L	1	2.39E-11	0.0294	0.1156	0.4307
NP_001019820.1 (CANX, hsa-circ-0001565)	Y	TIMFGPDK	C	2	1.57E-10	0.0057	0.0801	0.3834
NP_001019820.1 (CANX, hsa-circ-0001565)	Y	TIMFGPDKCGEDYK	L	2	2.81E-16	0.0163	0.0984	0.2856
NP_009055.2 (UTRN, hsa-circ-0001646)	K	LSQADGSFLK	E	1	3.58E-13	0.0025	0.0712	2.0321
NP_009055.2 (UTRN, hsa-circ-0001646)	K	LSQADGSFLKEK	L	1	1.74E-15	0.0011	0.0664	1.8006
NP_009055.2 (UTRN, hsa-circ-0001646)	K	QPDVILEASGPEAIQIR	D	2	7.65E-17	0.0306	0.117	0.4872
NP_009055.2 (UTRN, hsa-circ-0001646)	R	DTLTQLNAK	W	3	2.27E-12	0.0033	0.0749	1.1412
NP_009055.2 (UTRN, hsa-circ-0001646)	R	SSLLPTDYLVEINK	I	3	1.13E-15	0.0059	0.0804	1.3899
NP_009055.2 (UTRN, hsa-circ-0001646)	R	WDAIVAEVK	D	2	6.94E-13	0.0069	0.0827	1.7158
NP_009055.2 (UTRN, hsa-circ-0001648)	K	ECPIVGFR	Y	1	2.07E-11	0.0062	0.0812	1.5573
NP_009055.2 (UTRN, hsa-circ-0001648)	K	EVAGPTEM.*CDQR	Q	3	1.32E-13	0.0246	0.1098	0.8967
NP_009055.2 (UTRN, hsa-circ-0001648)	K	EVAGPTEMCDQR	Q	1	4.02E-17	0.0014	0.0667	1.7357
NP_009055.2 (UTRN, hsa-circ-0001648)	K	IGLMSLSK	G	2	1.83E-10	0.0021	0.0695	1.2353
NP_009055.2 (UTRN, hsa-circ-0001648)	K	LNQNDQLLSVPDVINCLTTTYDGLEQM.*HK	D	1	1.49E-13	0.0995	0.1725	0.3068
NP_009055.2 (UTRN, hsa-circ-0001648)	K	LNQNDQLLSVPDVINCLTTTYDGLEQMHK	D	1	9.16E-18	0.2836	0.2722	0.4322
NP_009055.2 (UTRN, hsa-circ-0001648)	R	QLGEVAAFGGSNIEPSVR	S	2	1.31E-18	0.1164	0.1834	0.2616
NP_009055.2 (UTRN, hsa-circ-0001648)	R	QLGLLLHDAIQIPR	Q	2	1.14E-14	0.0508	0.1367	0.3757
NP_009055.2 (UTRN, hsa-circ-0001648)	R	SCFQQNNNKPEISVK	E	3	4.14E-20	0.0004	0.0678	1.4018
NP_004902.1 (PDIA4, hsa_circ_0001766)	K	DKDPPIPVAK	I	5	1.34E-13	0.0065	0.0818	0.5903
NP_004902.1 (PDIA4, hsa_circ_0001766)	K	GQAVDYEGSR	T	4	4.35E-14	0.0089	0.0869	0.5847
NP_004902.1 (PDIA4, hsa_circ_0001766)	K	GQAVDYEGSRTQEEIVAK	V	3	2.54E-23	0.0028	0.0727	0.326
NP_004902.1 (PDIA4, hsa_circ_0001766)	K	IANILKDKDPPIPVAK	I	6	2.47E-19	0.0331	0.1194	0.3999
NP_004902.1 (PDIA4, hsa_circ_0001766)	K	IDATSASVLASR	F	9	3.95E-15	0.0139	0.0945	0.1322
NP_004902.1 (PDIA4, hsa_circ_0001766)	K	KGQAVDYEGSR	T	5	2.52E-15	0.0109	0.0902	0.6739
NP_004902.1 (PDIA4, hsa_circ_0001766)	K	KGQAVDYEGSRTQEEIVAK	V	3	3.21E-18	0.0102	0.0887	0.3606

NP_004902.1 (PDIA4, hsa_circ_0001766)	K	QFAPEYEK	I	6	6.97E-12	0.0051	0.0783	0.4683
NP_001104567.1 (LYN, hsa-circ-0001803)	K	DAWEIPR	E	1	4.35E-10	0.0076	0.0842	-0.1558
NP_001104567.1 (LYN, hsa-circ-0001803)	R	SLDNGGYYISPR	I	1	3.04E-16	0.0159	0.0979	1.8912
NP_006175.2 (NUCB1, hsa-circ-0002084)	K	EVWEELDGLDPNR	F	1	2.53E-16	0.0274	0.1135	0.5057
NP_006175.2 (NUCB1, hsa-circ-0002084)	K	EVWEELDGLDPNRFNPK	T	3	3.11E-17	0.105	0.176	0.3186
NP_006175.2 (NUCB1, hsa-circ-0002084)	K	VNVPGSQAQLK	E	4	6.78E-15	0.0128	0.093	0.3431
NP_006175.2 (NUCB1, hsa-circ-0002084)	K	VNVPGSQAQLKEVWEELDGLDPNRFNPK	T	1	2.02E-10	0.2112	0.2368	0.1171
NP_006175.2 (NUCB1, hsa-circ-0002084)	R	DLAQYDAAHHEEF	K	1	1.1E-13	0.1055	0.1764	-0.2053
NP_006175.2 (NUCB1, hsa-circ-0002084)	R	DLAQYDAAHHEEFKR	Y	3	2.82E-15	0.2182	0.2403	0.2982
NP_006175.2 (NUCB1, hsa-circ-0002084)	R	KLEEQQR	R	1	6.67E-10	0.1056	0.1765	0.4093
NP_006175.2 (NUCB1, hsa-circ-0002084)	R	KLEEQQRR	H	1	5.19E-10	0.0835	0.1612	0.6606
NP_006175.2 (NUCB1, hsa-circ-0002084)	R	YEM.*LKEHER	R	2	6.75E-12	0.2138	0.2382	0.3435
NP_006175.2 (NUCB1, hsa-circ-0002084)	R	YLESLGEEQRK	E	2	1.59E-13	0.3409	0.2984	0.234
NP_006175.2 (NUCB1, hsa-circ-0002084)	R	YLESLGEEQRKEAER	K	1	2.7E-13	0.2987	0.279	0.2162
NP_057525.1 (CRIM1, hsa-circ-0002346)	R	LTADGCCTLPTR	C	1	4.1E-16	0.0061	0.0812	1.2941
NP_057525.1(CRIM1, hsa-circ-0002348)	R	LTADGCCTLPTR	C	1	4.1E-16	0.0061	0.0812	1.2941
NP_037458.3 (RMC1, hsa-circ-0002757)	K	FEIELPAAPK	S	2	4.82E-13	0.181	0.221	0.1027
NP_057525.1(CRIM1, hsa-circ-0003578)	R	LTADGCCTLPTR	C	1	4.1E-16	0.0061	0.0812	1.2941
NP_612411.4 (SP140L, hsa-circ-0003922)	R	VVYNVLSELEK	T	1	6.27E-13	0.0125	0.0926	1.5296
NP_061176.3 (DHTKD1. hsa-circ-0004630)	K	DVIIDLLCYR	Q	2	2.18E-13	0.1163	0.1833	0.1571
NP_061176.3 (DHTKD1. hsa-circ-0004630)	K	LM.*LESQEFDHFLATK	F	1	2.08E-15	0.9258	0.5031	-0.0044
NP_061176.3 (DHTKD1. hsa-circ-0004630)	K	LMLESQEFDHFLATK	F	1	3.66E-19	0.2583	0.2602	0.1302
NP_061176.3 (DHTKD1. hsa-circ-0004630)	K	RFEELQKETFTTEER	K	1	1.64E-14	0.5693	0.3903	0.026
NP_061176.3 (DHTKD1. hsa-circ-0004630)	R	KDVIIDLLCYR	Q	1	1.41E-13	0.0101	0.0888	0.1025
NP_061176.3 (DHTKD1. hsa-circ-0004630)	R	LAFEYQR	Q	2	2.13E-10	0.05	0.1361	0.3686
NP_061176.3 (DHTKD1. hsa-circ-0004630)	R	QDGDYSPDNSAQPGDR	V	3	7.94E-18	0.0987	0.1719	0.2884
NP_061176.3 (DHTKD1. hsa-circ-0004630)	R	QWGHNELDEPFYTNPIM.*YK	I	2	2.19E-20	0.2355	0.249	0.0686
NP_061176.3 (DHTKD1. hsa-circ-0004630)	R	SSLYCSDIGK	L	3	1.43E-13	0.1302	0.1919	0.2057
NP_061176.3 (DHTKD1. hsa-circ-0004630)	R	YGGEGAESMM.*GFFHELLK	M	1	3.9E-19	0.6368	0.4144	0.0428
NP_061176.3 (DHTKD1. hsa-circ-0004630)	R	YGGEGAESMMGFFHELLK	M	1	1.92E-22	0.7548	0.4528	0.0653
NP_057525.1 (CRIM1, hsa-circ-0005442)	R	LTADGCCTLPTR	C	1	4.1E-16	0.0061	0.0812	1.2941
NP_006323.2 (IFI30, hsa-circ-0005571)	K	CQHGEEECKFNK	V	2	2.66E-15	0.014	0.0948	1.436
NP_006323.2 (IFI30, hsa-circ-0005571)	K	TGNLYLR	G	3	4.41E-10	0.004	0.076	1.1569
NP_057525.1 (CRIMA1, hsa-circ-0005579)	R	LTADGCCTLPTR	C	1	4.1E-16	0.0061	0.0812	1.2941
NP_001137451.1 (NDE1, hsa-circ-0005869)	R	ELEQANDDLER	A	1	1.42E-14	0.1847	0.2229	-0.139
NP_001137451.1 (NDE1, hsa-circ-0005869)	R	ISALNIVGDLLR	K	4	1.51E-14	0.6487	0.4184	0.0457
NP_001020750.1 (NDEL1, hsa-circ-0005869)	K	GTENTFPSPK	A	1	4.24E-14	0.6661	0.424	0.0354
NP_001020750.1 (NDEL1, hsa-circ-0005869)	K	QVSVLEDDLSQTR	A	1	9.94E-17	0.0938	0.1685	0.0346
NP_001020750.1 (NDEL1, hsa-circ-0005869)	R	ATIVSLEDFEQR	L	2	1.06E-15	0.1124	0.1808	0.1841
NP_055907.3 (SMG1, hsa-circ-0006434)	K	GLEDLHLDER	I	1	3.89E-13	0.0308	0.1172	0.2503
NP_055907.3 (SMG1, hsa-circ-0006434)	K	WFQDNYGDAIENALEK	L	3	3.83E-22	0.5296	0.3757	-0.0082
NP_055907.3 (SMG1, hsa-circ-0006434)	R	LEEISPWLAAMTNTEIALPGEVSAR	D	1	3.37E-22	0.4133	0.329	-0.2262
NP_055907.3 (SMG1, hsa-circ-0006434)	R	SGLIQWVDGATPLFGLYK	R	1	2.68E-13	0.6365	0.4144	-0.1654
NP_055907.3 (SMG1, hsa-circ-0006434)	R	SITAAPAETPHEK	W	1	1.4E-15	0.8582	0.4841	-0.0346
NP_001004060.1 (NOMO2, hsa-circ-0006735)	R	EDGSFSFYSLPSGGYTVIPFYR	G	1	1.73E-19	0.6212	0.4092	-0.0344
NP_001139021.1 (TXNDC5. hsa-circ-0007250)	K	FYAPWCGHCK	T	1	4.43E-16	0.587	0.397	-0.0217
NP_005733.1 (PDIA6, hsa-circ-0007250)	K	DVIELTDDSFDK	N	4	8.2E-16	0.0275	0.1136	0.3272
NP_005733.1 (PDIA6, hsa-circ-0007250)	R	SGGYSSGK	Q	1	2.86E-11	0.0437	0.1303	0.2503
NP_005733.1 (PDIA6, hsa-circ-0007250)	R	TGEAIVDAALSALR	Q	17	2.7E-17	0.0022	0.0702	0.2905
NP_689799.3 (USP54. hsa-circ-0018818)	R	ECGEDEQYSAENLRR	I	1	8.94E-11	0.5915	0.3986	-0.0489
NP_998811.1 (WARS, hsa-circ-0033184)	K	AAAGEDYK	A	1	1.39E-11	0.0012	0.0658	3.4449
NP_998811.1 (WARS, hsa-circ-0033184)	K	AAAGEDYKADCPPGN	P	1	5.95E-19	0.0043	0.0765	1.9094

NP_998811.1 (WARS, hsa-circ-0033184)	K	AAAGEDYKADCPPGNPAPTSNHGPDATEAEEDFVDPWTVQTSSAK	G	3	8.92E-31	0.0006	0.0675	2.871
NP_998811.1 (WARS, hsa-circ-0033184)	K	ADCPPGNPAPTSNHGPDATEAEEDFVDPWTVQTSSAK	G	1	6.06E-14	0.078	0.1577	1.7501
NP_998811.1 (WARS, hsa-circ-0033184)	K	DIIACGFDINK	T	3	5.91E-15	0.0001	0.0671	2.8485
NP_998811.1 (WARS, hsa-circ-0033184)	K	DLTLDQAYSYAVENAK	D	13	2.56E-21	0.0011	0.0667	1.9368
NP_998811.1 (WARS, hsa-circ-0033184)	K	DLTLDQAYSYAVENAKDIIACGFDINK	T	5	8.54E-28	0.0014	0.0666	3.2025
NP_998811.1 (WARS, hsa-circ-0033184)	K	ELINRIER	A	2	1.98E-10	0.0007	0.0669	2.2167
NP_998811.1 (WARS, hsa-circ-0033184)	K	GIDYDKLIVR	F	10	2.66E-13	0.0021	0.0696	3.9177
NP_998811.1 (WARS, hsa-circ-0033184)	K	IDKELINR	I	5	1.38E-10	0.0007	0.0664	3.3177
NP_998811.1 (WARS, hsa-circ-0033184)	K	IDKELINRIER	A	9	9.64E-14	0.0055	0.0798	3.7701
NP_998811.1 (WARS, hsa-circ-0033184)	K	KPFYLYTGR	G	1	1.75E-12	0.0025	0.0717	2.4857
NP_998811.1 (WARS, hsa-circ-0033184)	K	WLQDVFNVPLVIQM.*TDDEK	Y	5	3.18E-19	0.0027	0.0725	0.9593
NP_998811.1 (WARS, hsa-circ-0033184)	K	WLQDVFNVPLVIQM.*TDDEKYLWK	D	2	3.04E-15	0.021	0.1052	0.5641
NP_998811.1 (WARS, hsa-circ-0033184)	K	WLQDVFNVPLVIQMTDDEK	Y	6	8.74E-26	0.0026	0.0714	1.9547
NP_998811.1 (WARS, hsa-circ-0033184)	R	ATGQRPH	H	3	1.23E-10	0.0003	0.0666	1.8936
NP_998811.1 (WARS, hsa-circ-0033184)	R	DM.*NQVLDAYENK	K	3	6.85E-16	0.0021	0.0696	1.0813
NP_998811.1 (WARS, hsa-circ-0033184)	R	DM.*NQVLDAYENKKPFYLYTGR	G	1	7.03E-10	0.0022	0.0701	2.9624
NP_998811.1 (WARS, hsa-circ-0033184)	R	DMNQVLDAYENK	K	6	2.43E-17	0.0025	0.0714	1.358
NP_998811.1 (WARS, hsa-circ-0033184)	R	DMNQVLDAYENKKPF	Y	4	7.43E-17	0.0011	0.0669	3.0821
NP_998811.1 (WARS, hsa-circ-0033184)	R	DMNQVLDAYENKKPFYLYTGR	G	11	3.75E-21	0.0014	0.0669	3.5379
NP_998811.1 (WARS, hsa-circ-0033184)	R	FGSSKIDKELINR	I	1	2.95E-12	0.0014	0.0668	2.3433
NP_998811.1 (WARS, hsa-circ-0033184)	R	GIFFSHR	D	4	1.68E-10	0.0006	0.0678	2.7199
NP_998811.1 (WARS, hsa-circ-0033184)	R	GPSSEAMHVGHLIPFIFTK	W	1	3.51E-23	0.0082	0.0859	1.7589
NP_998811.1 (WARS, hsa_circ_0033191)	K	AAAGEDYK	A	1	1.39E-11	0.0012	0.0658	3.4449
NP_998811.1 (WARS, hsa_circ_0033191)	K	AAAGEDYKADCPPGN	P	1	5.95E-19	0.0043	0.0765	1.9094
NP_998811.1 (WARS, hsa_circ_0033191)	K	AAAGEDYKADCPPGNPAPTSNHGPDATEAEEDFVDPWTVQTSSAK	G	3	8.92E-31	0.0006	0.0675	2.871
NP_998811.1 (WARS, hsa_circ_0033191)	K	ADCPPGNPAPTSNHGPDATEAEEDFVDPWTVQTSSAK	G	1	6.06E-14	0.078	0.1577	1.7501
NP_998811.1 (WARS, hsa_circ_0033191)	K	ELINRIER	A	2	1.98E-10	0.0007	0.0669	2.2167
NP_998811.1 (WARS, hsa_circ_0033191)	K	GIDYDKLIVR	F	10	2.66E-13	0.0021	0.0696	3.9177
NP_998811.1 (WARS, hsa_circ_0033191)	K	IDKELINR	I	5	1.38E-10	0.0007	0.0664	3.3177
NP_998811.1 (WARS, hsa_circ_0033191)	K	IDKELINRIER	A	9	9.64E-14	0.0055	0.0798	3.7701
NP_998811.1 (WARS, hsa_circ_0033191)	R	ATGQRPH	H	3	1.23E-10	0.0003	0.0666	1.8936
NP_998811.1 (WARS, hsa_circ_0033191)	R	FGSSKIDKELINR	I	1	2.95E-12	0.0014	0.0668	2.3433
NP_998811.1 (WARS, hsa_circ_0033191)	R	GIFFSHR	D	4	1.68E-10	0.0006	0.0678	2.7199
NP_998827.1 (STAT3, hsa-circ-0043812)	K	RGLSIEQLTTLAEK	L	1	2.83E-14	0.0221	0.1065	0.9026
NP_998827.1 (STAT3, hsa-circ-0043812)	R	GLSIEQLTTLAEK	L	4	7.3E-16	0.0034	0.0748	1.1574
NP_998827.1 (STAT3, hsa-circ-0043815)	R	FLQESNVLYQHNLR	R	1	4.88E-15	0.2577	0.2599	0.2153
NP_998827.1 (STAT3, hsa-circ-0043815)	R	QFLAPWIESQDWAYAASK	E	2	1.52E-24	0.0007	0.0653	1.1468
NP_998827.1 (STAT3, hsa-circ-0043815)	R	YLEKPM.*EIAR	I	1	4.6E-11	0.0229	0.1075	1.0385
NP_998827.1 (STAT3, hsa-circ-0043815)	R	YLEKPMEIAR	I	2	6.63E-11	0.0013	0.0661	1.3642
NP_998827.1 (STAT3, hsa-circ-0043816)	R	FLQESNVLYQHNLR	R	1	4.88E-15	0.2577	0.2599	0.2153
NP_998827.1 (STAT3, hsa-circ-0043816)	R	QFLAPWIESQDWAYAASK	E	2	1.52E-24	0.0007	0.0653	1.1468
NP_998827.1 (STAT3, hsa-circ-0043816)	R	YLEKPM.*EIAR	I	1	4.6E-11	0.0229	0.1075	1.0385
NP_998827.1 (STAT3, hsa-circ-0043816)	R	YLEKPMEIAR	I	2	6.63E-11	0.0013	0.0661	1.3642
NP_057525.1 (CRIM1, hsa-miR-0053955)	R	LTADGCCTLPTR	C	1	4.1E-16	0.0061	0.0812	1.2941
NP_057525.1 (CRIM1, hsa-circ-0053958)	R	LTADGCCTLPTR	C	1	4.1E-16	0.0061	0.0812	1.2941
NP_057525.1 (CRIM1, hsa-circ-0053967)	R	LTADGCCTLPTR	C	1	4.1E-16	0.0061	0.0812	1.2941
NP_002345.2 (EPCAM, hsa-circ-0057360)	K	AEIKEM.*GEM.*HR	E	4	1.58E-14	0.103	0.1749	-0.2493
NP_002345.2 (EPCAM, hsa-circ-0057360)	K	AEIKEM.*GEMHR	E	4	2.93E-15	0.2437	0.253	-0.1529
NP_002345.2 (EPCAM, hsa-circ-0057360)	K	AEIKEMGEM.*HR	E	2	3.12E-15	0.1906	0.226	-0.1069
NP_002345.2 (EPCAM, hsa-circ-0057360)	K	AEIKEMGEMH	R	1	1.93E-14	0.0373	0.1239	0.4995
NP_002345.2 (EPCAM, hsa-circ-0057360)	K	AEIKEMGEMHR	E	2	1.09E-15	0.2431	0.2527	-0.1628
NP_002345.2 (EPCAM, hsa-circ-0057360)	K	EM.*GEM.*H	R	1	4.64E-10	0.1528	0.2052	1.2842

NP_002345.2 (EPCAM, hsa-circ-0057360)	K	EM.*GEM.*HR	E	10	7.03E-11	0.6154	0.4072	-0.0591
NP_002345.2 (EPCAM, hsa-circ-0057360)	K	EM.*GEMHR	E	5	2.62E-11	0.8001	0.4672	0.0564
NP_002345.2 (EPCAM, hsa-circ-0057360)	K	EMGEM.*HR	E	5	2.62E-11	0.071	0.1527	0.1426
NP_002345.2 (EPCAM, hsa-circ-0057360)	K	EMGEMHR	E	5	2.62E-11	0.9708	0.515	0.0093
NP_002345.2 (EPCAM, hsa-circ-0057360)	K	EMGEMHRELNA	-	1	6.83E-16	0.6562	0.4208	0.0387
NP_006819.2 (ASCC3, hsa-circ-0077495)	K	AGEDLEVSEGLM.*CFDPK	E	1	2.3E-18	0.3331	0.2948	-0.0434
NP_006819.2 (ASCC3, hsa-circ-0077495)	K	AGEDLEVSEGLMCFDPK	E	1	6.03E-22	0.1424	0.1993	0.1537
NP_006819.2 (ASCC3, hsa-circ-0077495)	K	ILGENAKPNYGCQVTIQSEQEK	Q	3	4.75E-27	0.7463	0.4502	0.0211
NP_006819.2 (ASCC3, hsa-circ-0077495)	K	MILPEGIQR	E	2	4.48E-11	0.2486	0.2555	0.2115
NP_006819.2 (ASCC3, hsa-circ-0077495)	K	TSAFIAGAK	M	1	3.35E-12	0.8108	0.4705	0.0144
NP_006819.2 (ASCC3, hsa-circ-0077495)	R	ENNKLYEEVR	I	1	4.28E-14	0.1693	0.2147	0.0828
NP_006819.2 (ASCC3, hsa-circ-0077495)	R	EQALLNAR	S	2	8.02E-11	0.9225	0.5022	0.0202
NP_006819.2 (ASCC3, hsa-circ-0077495)	R	FQALQDNCK	K	1	6.72E-12	0.8094	0.4701	0.0149
NP_055205.2 (SND1, hsa-circ-0082141)	K	KVNVTVDYIRPASPATETVPAFSER	T	5	2.62E-31	0.7818	0.4613	0.0073
NP_055205.2 (SND1, hsa-circ-0082141)	K	LRPLYDIPYM.*FEAR	E	3	6.27E-18	0.1912	0.2263	0.1565
NP_055205.2 (SND1, hsa-circ-0082141)	K	LRPLYDIPYMFEAR	E	3	5.5E-19	0.3353	0.2958	0.0863
NP_055205.2 (SND1, hsa-circ-0082141)	K	VNVTVDYIRPASPATETVPAFSER	T	8	2.35E-22	0.1605	0.2097	0.0504
NP_055205.2 (SND1, hsa-circ-0082141)	R	SSHYDELLAAEAR	A	5	5.38E-17	0.1097	0.1791	0.0105
NP_055205.2 (SND1, hsa-circ-0082141)	R	YRQDDDQR	S	2	2.56E-12	0.8203	0.4733	-0.0181
NP_056989.2 (TRIM24, hsa-circ-0082580)	K	FTGNQIQNR	I	2	3.51E-13	0.0562	0.1417	-0.3015
NP_056989.2 (TRIM24, hsa-circ-0082580)	K	LMQQQQEVAGLSK	Q	2	2.49E-17	0.0972	0.171	-0.236
NP_056989.2 (TRIM24, hsa-circ-0082580)	K	LYCETCDK	L	1	2.05E-10	0.5586	0.3864	-0.1157
NP_056989.2 (TRIM24, hsa-circ-0082580)	K	LYCETCDKLTCR	D	2	1.6E-16	0.0231	0.1077	-0.2206
NP_056989.2 (TRIM24, hsa-circ-0082580)	K	VIIDTLITK	L	1	2.23E-11	0.0091	0.0871	-0.3022
NP_056989.2 (TRIM24, hsa-circ-0082580)	R	IIEVNQNQK	Q	2	1.77E-12	0.0237	0.1087	-0.1685
NP_056989.2 (TRIM24, hsa-circ-0082580)	R	YQFIEEAFQNQK	V	4	5.05E-17	0.0945	0.1689	-0.1478
NP_148980.2 (TRIM33, hsa-circ-0082580)	R	DCQLLEHK	E	1	1.29E-11	0.7271	0.4442	-0.0352
NP_148980.2 (TRIM33, hsa-circ-0082580)	R	DCQLLEHKEHR	Y	1	1.86E-11	0.0217	0.106	-0.3154
NP_073587.1 (PARP12, hsa-circ-0082688)	K	LGM.*SSDLVSR	L	1	1.94E-10	0.0008	0.0654	1.4766
NP_073587.1 (PARP12, hsa-circ-0082688)	K	LGMSSDLVSR	L	1	8.25E-12	0.0074	0.0838	0.8678
NP_073587.1 (PARP12, hsa-circ-0082688)	K	LHICQYFLQGECK	F	1	1.12E-17	0.0106	0.0896	0.254
NP_073587.1 (PARP12, hsa-circ-0082688)	K	SCSFQDK	C	1	8.47E-11	0.0041	0.076	1.2047
NP_073587.1 (PARP12, hsa-circ-0082688)	R	VPPLFVPQGTSER	K	1	5.83E-11	0.0145	0.0956	0.7357
NP_073587.1 (PARP12, hsa-circ-0082689)	K	LGM.*SSDLVSR	L	1	1.94E-10	0.0008	0.0654	1.4766
NP_073587.1 (PARP12, hsa-circ-0082689)	K	LGMSSDLVSR	L	1	8.25E-12	0.0074	0.0838	0.8678
NP_073587.1 (PARP12, hsa-circ-0082689)	K	LHICQYFLQGECK	F	1	1.12E-17	0.0106	0.0896	0.254
NP_073587.1 (PARP12, hsa-circ-0082689)	R	VPPLFVPQGTSER	K	1	5.83E-11	0.0145	0.0956	0.7357
NP_001104567.1 (LYN, hsa-circ-0084552)	K	GSLLDFLK	S	1	1.81E-10	0.0569	0.1423	0.3089
NP_001104567.1 (LYN, hsa-circ-0084552)	R	EEPIYIITEYMAK	G	1	3.74E-17	0.0028	0.0726	1.6941
NP_694592.1 (FYN, hsa-circ-0084552)	K	GSLLDFLKDGEGR	A	1	1.69E-15	0.8361	0.4778	0.0221
NP_938033.1 (SRC, hsa-circ-0084552)	K	GSLLDFLKGETGK	Y	1	5.55E-15	0.1129	0.1812	-0.1301
NP_005424.1 (YES1, hsa-circ-0084552)	K	GSLLDFLKEGDGK	Y	2	2.16E-15	0.2171	0.2397	0.1087
NP_056506.2 (WWWC3, hsa-circ-0089866)	K	EALLQELQLIIAQR	R	2	4.15E-15	0.6759	0.4273	0.0449
NP_001035089.1 (AFDN, hsa-circ-0089866)	K	ARLEEERER	K	1	1.47E-10	0.0164	0.0985	-0.1379

Table S6. Multi-species conserved sequences (MCS) analysis of the 84 differentially expressed circRNAs.

84 DE circRNAs	conserved in at least 5 species	At least 4 species	At least 2 species
Number	13 circRNAs	28 circRNAs	57 circRNAs
Percentage (%)	15.48%	33.33%	67.86%

Table S7. Predicted interactions between the two differentially expressed circWARS isoforms and miRNAs.

miRNA name	circWARS (CircBase ID)	Bound position (GRCh37)	clipExpNum	RBP	merClass
hsa-miR-192-5p	hsa_circ_0033184	chr14:100820807-100820829	16	AGO1,AGO1-4,AGO2	7mer-m8
hsa-miR-215-5p	hsa_circ_0033184	chr14:100820807-100820829	16	AGO1,AGO1-4,AGO2	7mer-m8
hsa-miR-212-5p	hsa_circ_0033184	chr14:100820087-100820111	13	AGO1-4,AGO2	8mer
hsa-miR-33a-5p	hsa_circ_0033184	chr14:100820816-100820836	12	AGO1,AGO1-4,AGO2	7mer-m8
hsa-miR-33b-5p	hsa_circ_0033184	chr14:100820816-100820835	12	AGO1,AGO1-4,AGO2	7mer-m8
hsa-miR-296-5p	hsa_circ_0033184	chr14:100820834-100820854	10	AGO1,AGO1-4,AGO2	7mer-m8
hsa-miR-423-5p	hsa_circ_0033184	chr14:100820831-100820853	10	AGO1,AGO1-4,AGO2	7mer-m8
hsa-miR-3184-5p	hsa_circ_0033184	chr14:100820831-100820854	10	AGO1,AGO1-4,AGO2	7mer-m8
hsa-miR-323b-3p	hsa_circ_0033184	chr14:100820772-100820793	9	AGO1,AGO1-4,AGO2	8mer
hsa-miR-425-5p	hsa_circ_0033184, hsa_circ_0033191	chr14:100828221-100828244	8	AGO1,AGO1-4,AGO2	7mer-m8
hsa-miR-3163	hsa_circ_0033184	chr14:100820854-100820875	7	AGO1-4,AGO2	7mer-m8
hsa-miR-21-5p	hsa_circ_0033184, hsa_circ_0033191	chr14:100828049-100828071	6	AGO1-4,AGO2	7mer-m8
hsa-miR-135a-5p	hsa_circ_0033184	chr14:100820859-100820881	6	AGO1-4,AGO2	7mer-m8
hsa-miR-135b-5p	hsa_circ_0033184	chr14:100820859-100820881	6	AGO1-4,AGO2	7mer-m8
hsa-miR-532-5p	hsa_circ_0033184, hsa_circ_0033191	chr14:100828064-100828085	6	AGO1-4,AGO2	8mer
hsa-miR-577	hsa_circ_0033184	chr14:100820852-100820872	6	AGO1-4,AGO2	7mer-m8
hsa-miR-590-5p	hsa_circ_0033184, hsa_circ_0033191	chr14:100828049-100828069	6	AGO1-4,AGO2	7mer-m8
hsa-miR-556-5p	hsa_circ_0033184, hsa_circ_0033191	chr14:100828046-100828067	5	AGO1-4,AGO2	7mer-m8
hsa-miR-642b-5p	hsa_circ_0033184, hsa_circ_0033191	chr14:100828159-100828180	2	AGO1-4	7mer-m8
hsa-miR-223-3p	hsa_circ_0033184	chr14:100820704-100820723	1	AGO1-4	8mer
hsa-miR-320a	hsa_circ_0033184	chr14:100820579-100820600	1	AGO1-4	7mer-m8
hsa-miR-345-5p	hsa_circ_0033184	chr14:100820571-100820592	1	AGO1-4	7mer-m8
hsa-miR-543	hsa_circ_0033184	chr14:100820730-100820751	1	AGO1-4	7mer-m8
hsa-miR-320b	hsa_circ_0033184	chr14:100820579-100820600	1	AGO1-4	7mer-m8
hsa-miR-320c	hsa_circ_0033184	chr14:100820579-100820598	1	AGO1-4	7mer-m8
hsa-miR-320d	hsa_circ_0033184	chr14:100820579-100820597	1	AGO1-4	7mer-m8
hsa-miR-4429	hsa_circ_0033184	chr14:100820579-100820598	1	AGO1-4	7mer-m8
hsa-miR-3064-5p	hsa_circ_0033184	chr14:100820567-100820589	1	AGO1-4	7mer-m8
hsa-miR-6504-5p	hsa_circ_0033184	chr14:100820567-100820586	1	AGO1-4	7mer-m8

circRNAs-miRNA interactions were predicted for the two isoforms of circWARS that were differentially expressed by intersecting the predicting target sites of miRNAs with binding sites of Ago protein derived from publicly available CLIP-seq data from StarBase (http://starbase.sysu.edu.cn/). The interactions of miRNA-circRNA were predicted by using miRanda program. The miRNAs are sorted based on highest clipExpNum (i.e the total number of Ago Clip-Seq experiments supporting the interaction). The miRNAs highlighted in red are predominantly expressed in isolated β-cells compared to human islets [37].

Supplementary Figures

Figure S1. Verification of circWARS, circEPSTI1, and circCRIM1 expression in EndoC-βH1 cells.

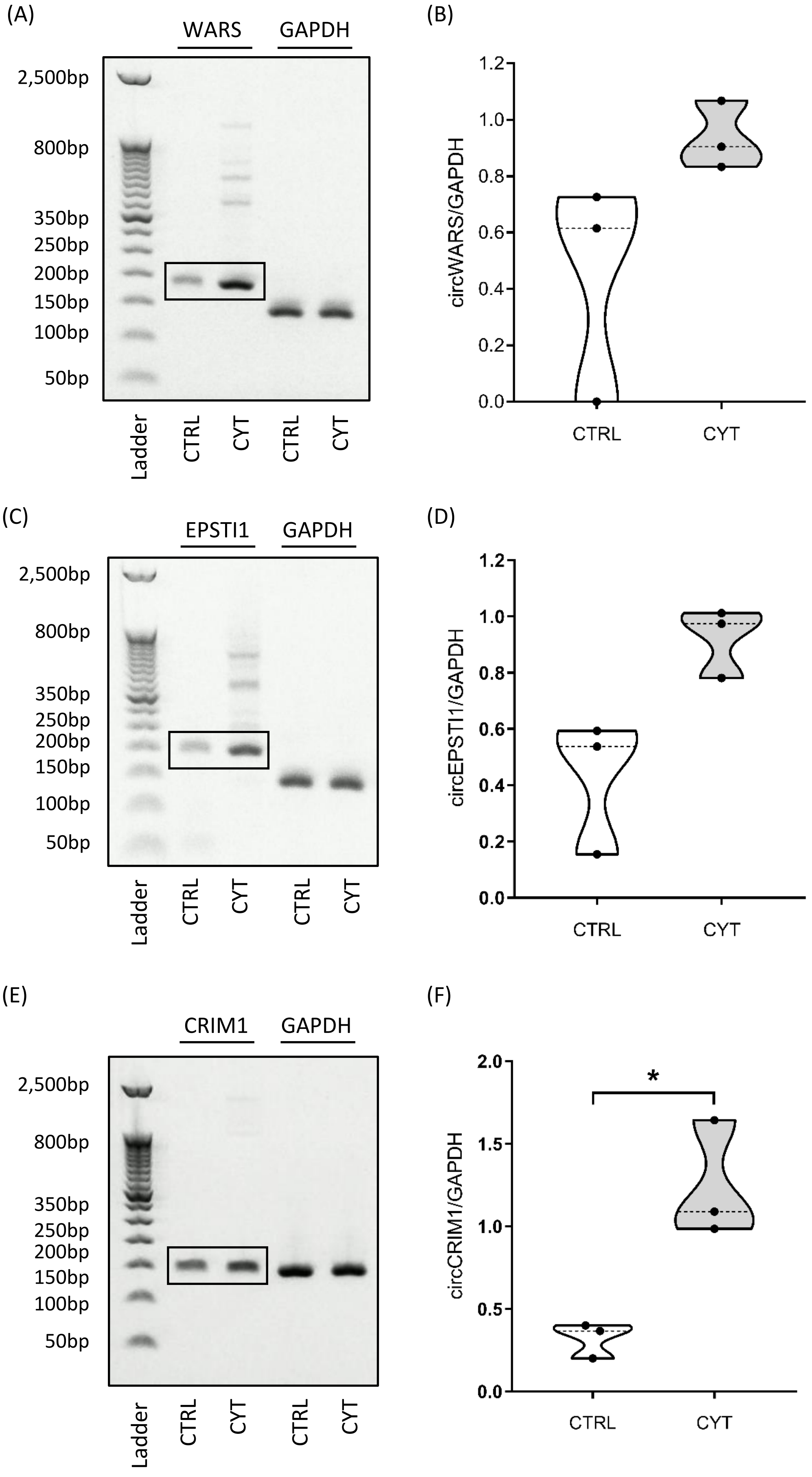

Gel electrophoresis visualization and quantification of circWARS (A-B), circEPSTI1 (C-D), and circCRIM1 (E-F) after real-time qPCR analysis on human EndoC-βH1 cells, left untreated (CTRL) or treated with IL-1β and IFN-γ (CYT) for 48 h, (n = 3). Quantification is shown as violin plots. *GAPDH* was used as housekeeping gene. * $p < 0.05$.

Figure S2. Verification of circWARS and circEPSTI1 expression in human islets.

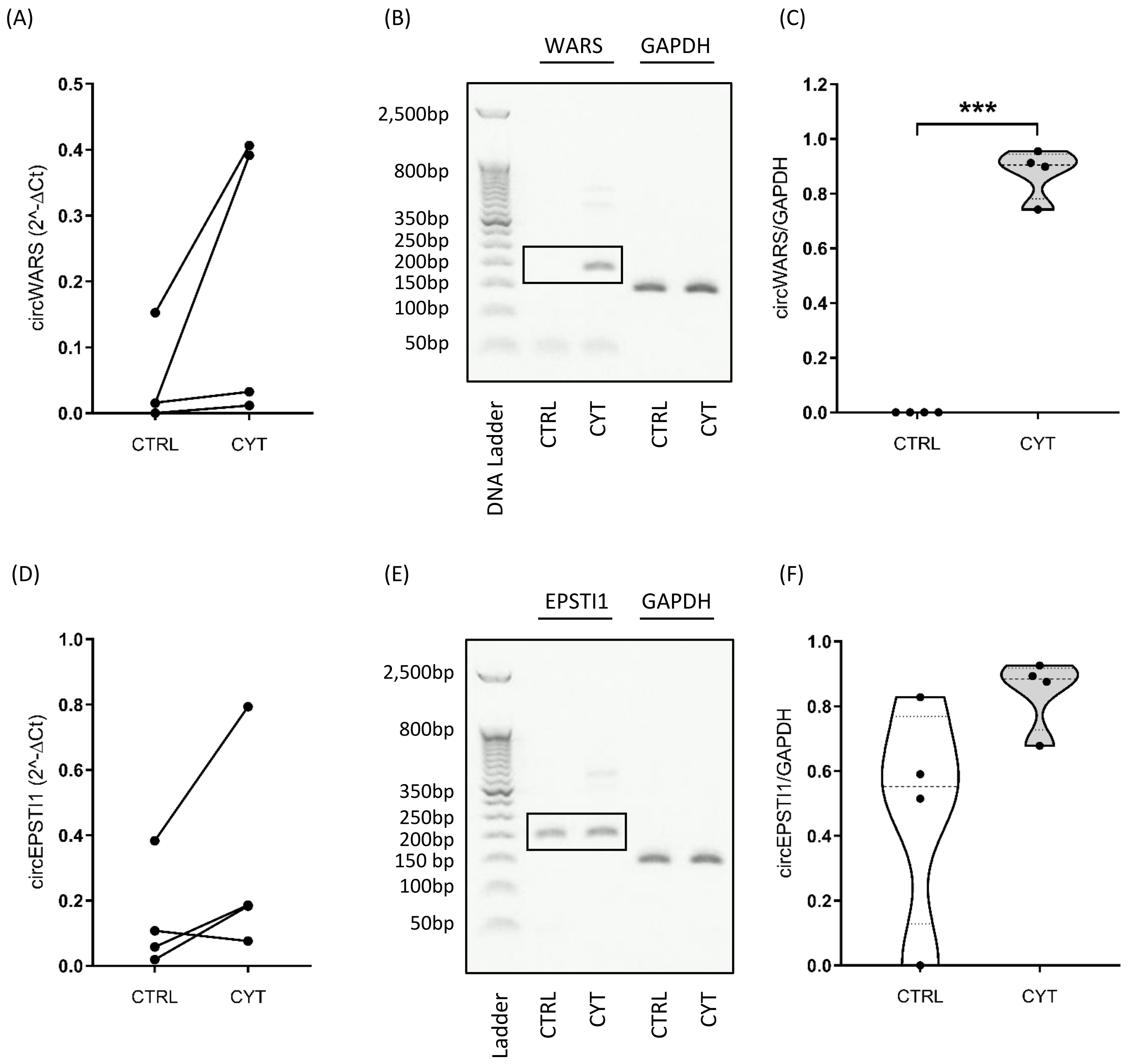

Gene expression levels of circWARS and circEPSTI1 were analyzed in isolated human pancreatic islets from 4 donors, left untreated (CTRL) or treated with IL-1β and IFN-γ (CYT) for 24 h, by real-time qPCR (A,D) and by gel electrophoresis visualization (B,E) and quantification (C,F) (n = 4). The normalized expression values are presented as 2^−ΔCt and quantified bands as violin plots. *GAPDH* was used as housekeeping gene. *** $p < 0.001$.

Figure S3. Sanger sequencing confirmed the expression of circWARS, circEPSTI1, and circCRIM1 in EndoC-βH1 cells.

(A)

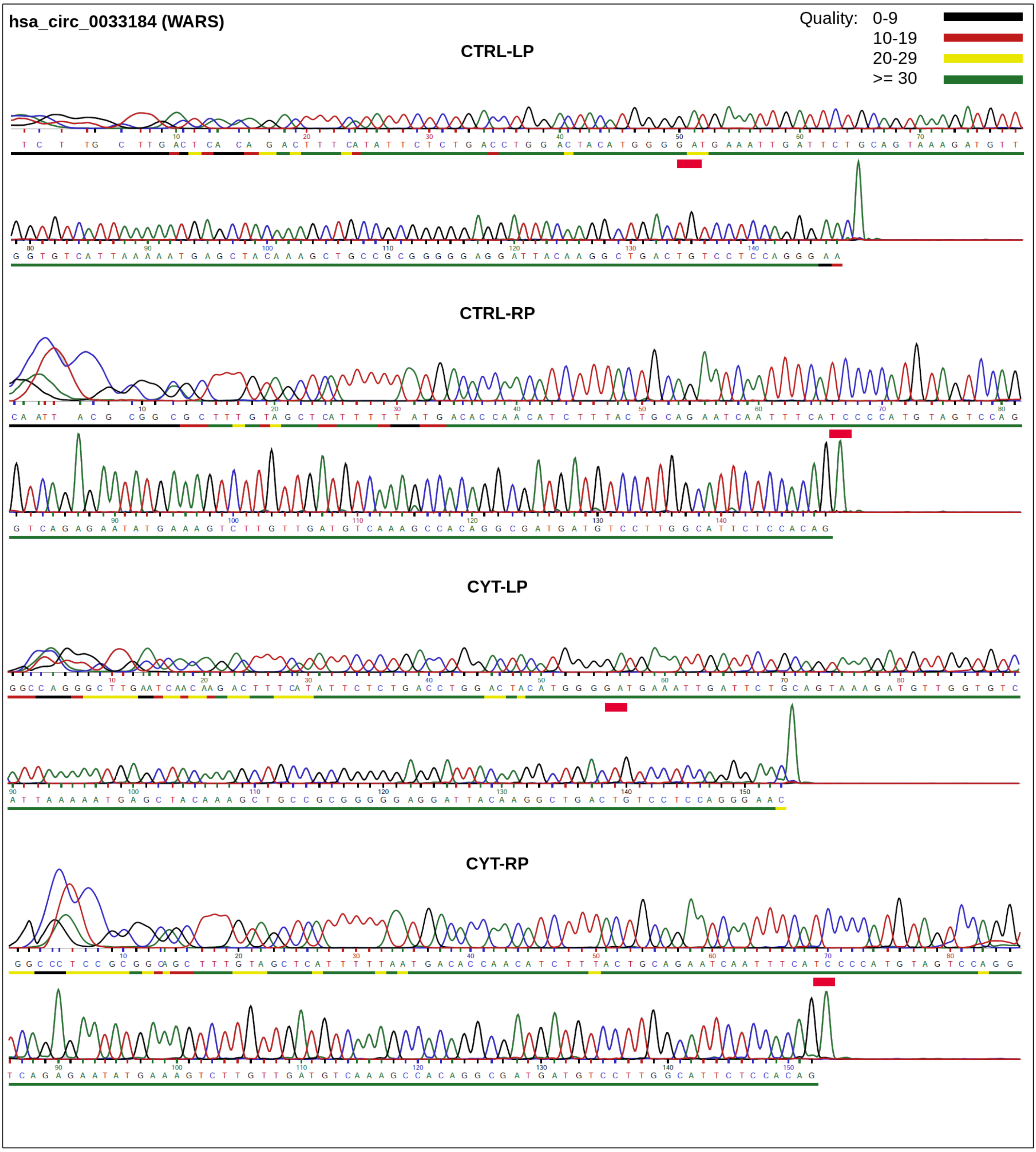

(B)

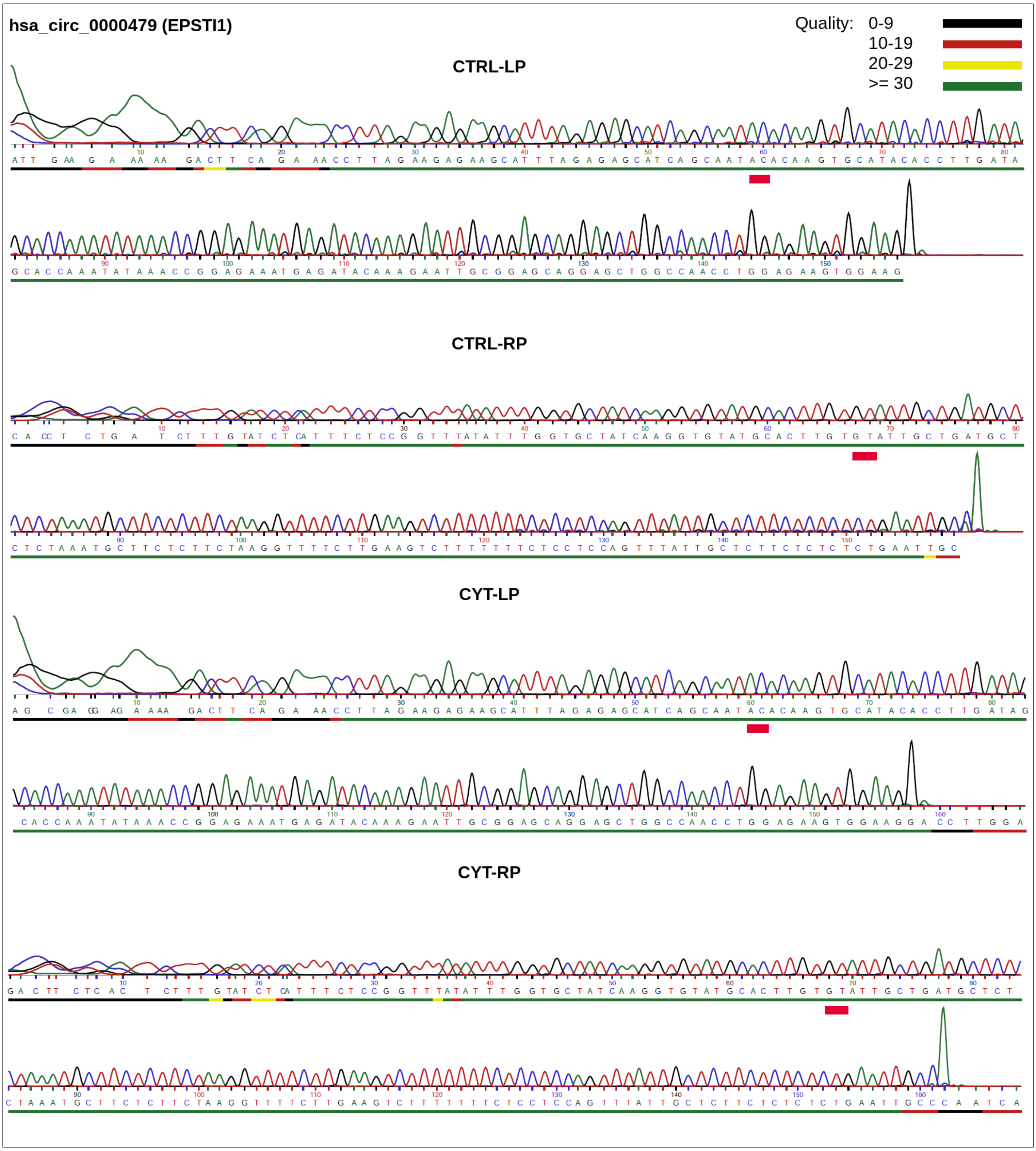
hsa_circ_0000479 (EPSTI1)
Quality: 0-9
10-19
20-29
>= 30
CTRL-LP
CTRL-RP
CYT-LP
CYT-RP

(C)

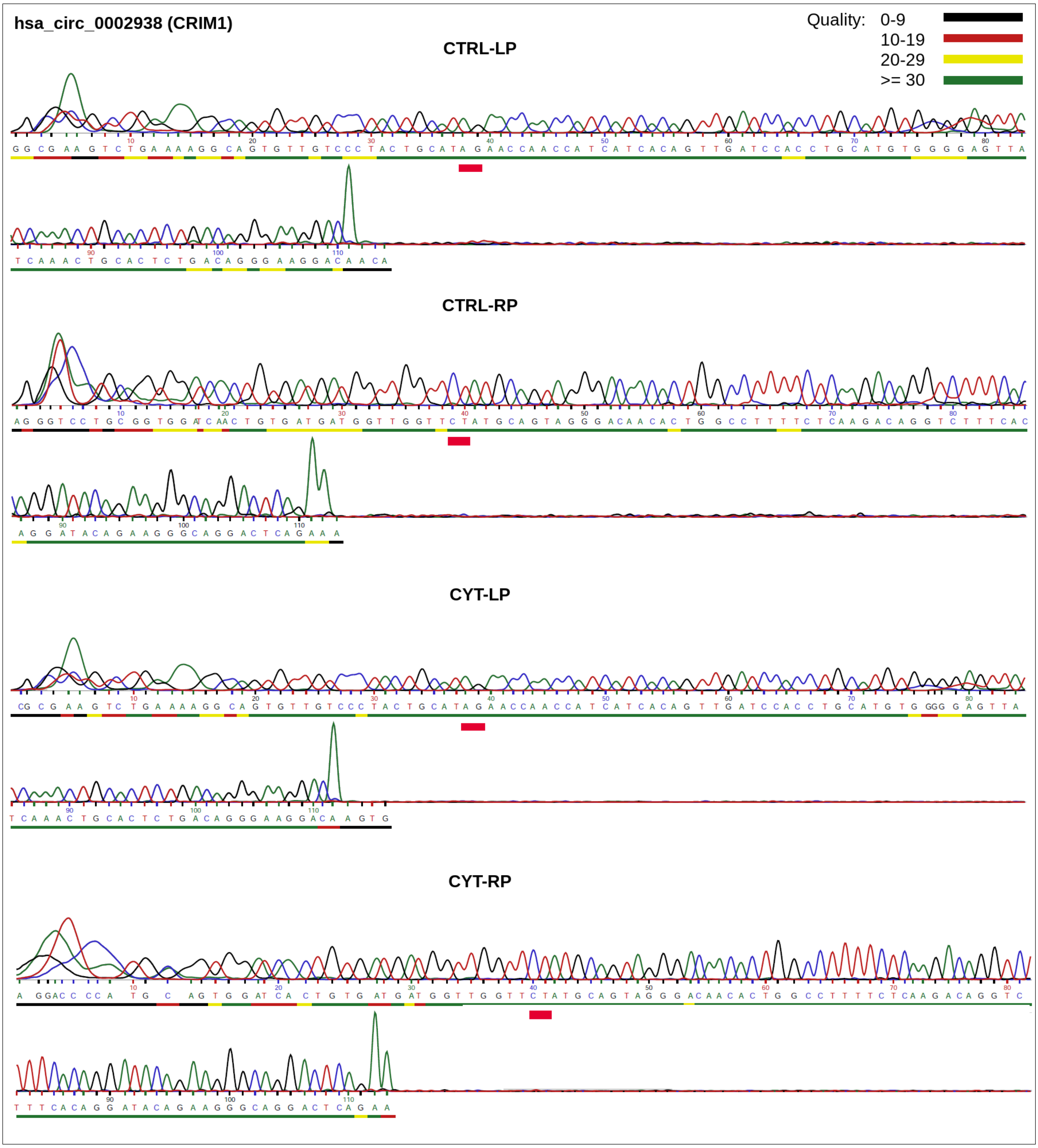

The figure shows four electropherogram from the PCR products of hsa_circ_0033184 (circWARS) (A), hsa_circ_0000479 (circEPSTI1) (B), and hsa_circ_0002938 (circCRIM1) (C). The back-splice junction is highlighted by the red bar under the sequence. CTRL-LP, CTRL-RP: control samples; CYT-LP, CYT-RP: cytokine-treated samples; LP: left primer; RP: right primer.

Figure S4. The expression of host genes WARS, EPSTI1, and CRIM1 in human islets.

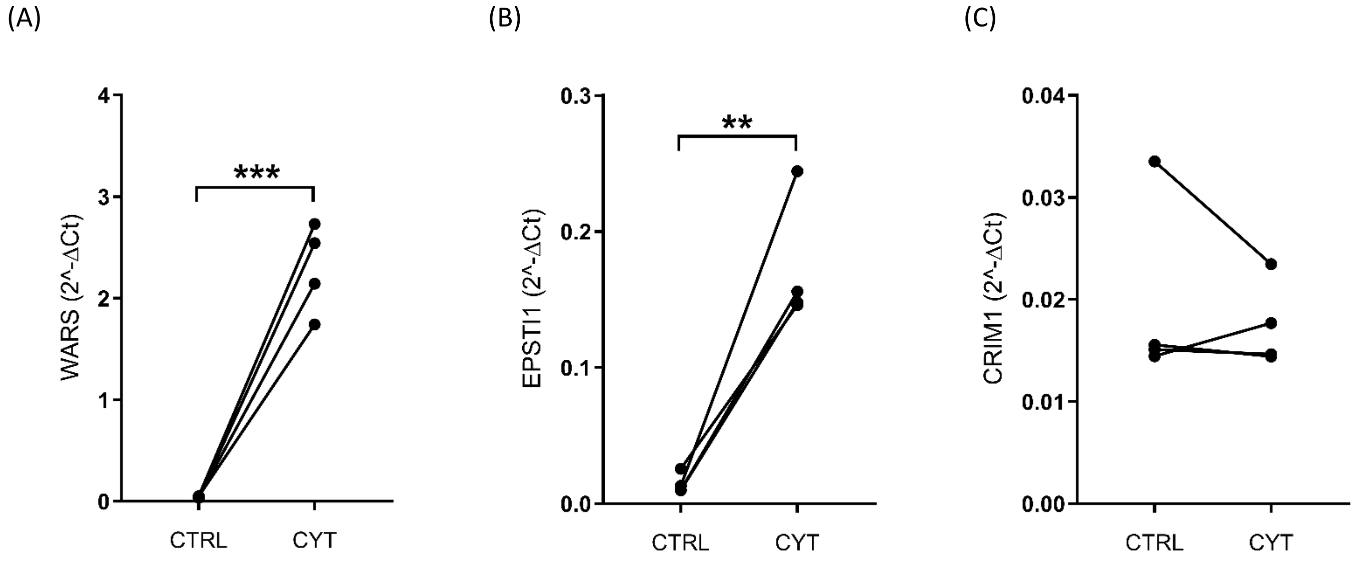

The mRNA levels of (A) WARS, (B) EPSTI1 and (C) CRIM1 were analyzed in isolated human pancreatic islets from 4 donors, left untreated (CTRL) or treated with IL-1β and IFN-γ (CYT) for 24 h. *GAPDH* was used as housekeeping gene. Data are presented as 2^−ΔCt, ** p<0.01, *** p<0.001.

Figure S5. The total number of RNA-binding protein (RBP) binding sites on junction sequences of circWARS, circEPSTI1, and circCRIM1.

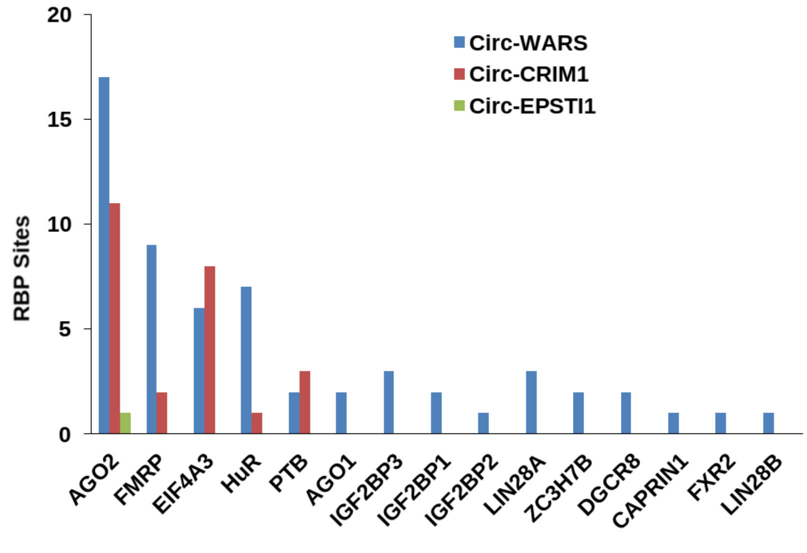

Figure S6. *CRIM1* and *EPSTI1* loci harbor multiple cytokine responsive elements

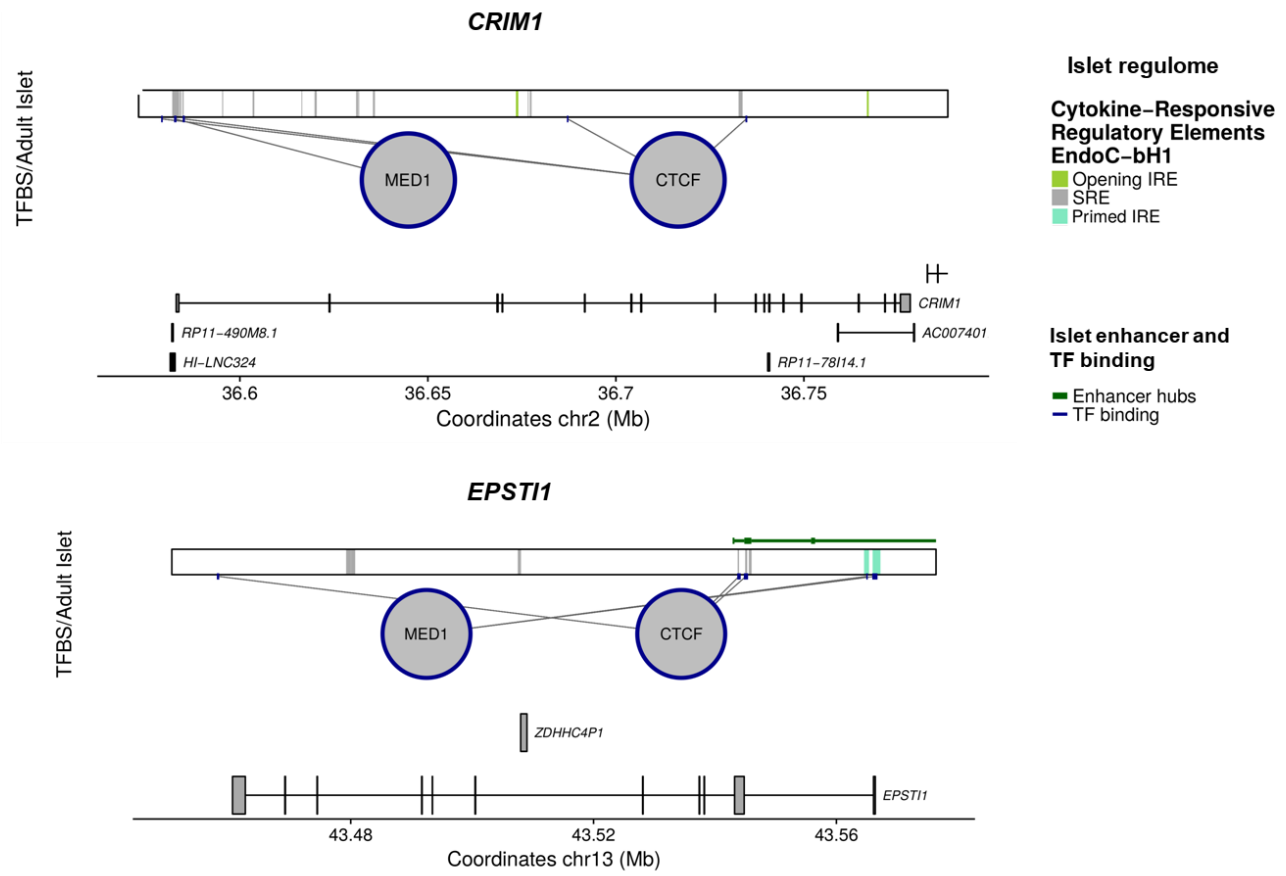

The 13 DE circRNAs isoforms at *CRIM1* locus harbor >100 SREs. Promoter of *EPSTI1* locus harbor primed IREs.

Figure S7. The expression of miRNA targets and miRNA levels in human islets.

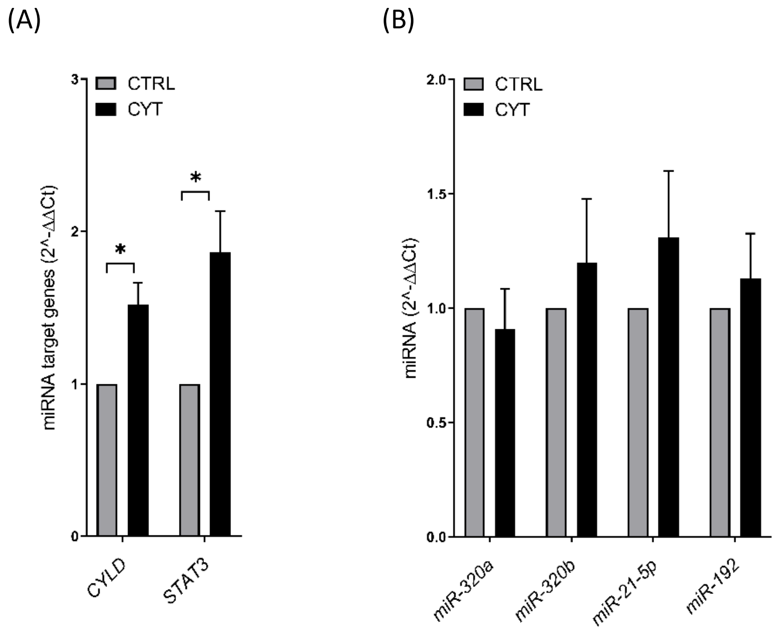

The expression level of (A) the miRNA target genes CYLD and STAT3 and (B) hsa-miR-192-5p, hsa-miR-21-5p and the hsa-miR-320 family in human islets after 24 h of cytokine exposure (n = 4). *GAPDH* and hsa-miR-375 was used as internal controls. Data are presented as 2^−ΔΔCt, * $p < 0.05$.
